# NKL homeobox gene activities in B-cell development and lymphomas

**DOI:** 10.1371/journal.pone.0205537

**Published:** 2018-10-11

**Authors:** Stefan Nagel, Roderick A. F. MacLeod, Corinna Meyer, Maren Kaufmann, Hans G. Drexler

**Affiliations:** Department of Human and Animal Cell Lines, Leibniz-Institute DSMZ–German Collection of Microorganisms and Cell Cultures, Braunschweig, Germany; University of South Alabama Mitchell Cancer Institute, UNITED STATES

## Abstract

Homeobox genes encode transcription factors which regulate basic processes in development and cell differentiation. Several members of the NKL subclass are deregulated in T-cell progenitors and support leukemogenesis. We have recently described particular expression patterns of nine NKL homeobox genes in early hematopoiesis and T-cell development. Here, we screened NKL homeobox gene activities in normal B-cell development and extended the NKL-code to include this lymphoid lineage. Analysis of public expression profiling datasets revealed that HHEX and NKX6-3 were the only members differentially active in naïve B-cells, germinal center B-cells, plasma cells and memory B-cells. Subsequent examination of different types of B-cell malignancies showed both aberrant overexpression of NKL-code members and ectopic activation of subclass members physiologically silent in lymphopoiesis including BARX2, DLX1, EMX2, NKX2-1, NKX2-2 and NKX3-2. Based on these findings we performed detailed studies of the B-cell specific NKL homeobox gene NKX6-3 which showed enhanced activity in patient subsets of follicular lymphoma, mantle cell lymphoma and diffuse large B-cell lymphoma (DLBCL), and in three DLBCL cell lines to serve as in vitro models. While excluding genomic and chromosomal rearrangements at the locus of NKX6-3 (8p11) promoter studies demonstrated that B-cell factors MYB and PAX5 activated NKX6-3 transcription. Furthermore, aberrant BMP7/SMAD1-signalling and deregulated expression of chromatin complex components AUTS2 and PCGF5 promoted NKX6-3 activation. Finally, NKL homeobox genes HHEX, HLX, MSX1 and NKX6-3 were expressed in B-cell progenitors and generated a regulatory gene network in cell lines which we propose may provide physiological support for NKL-code formation in early B-cell development. Together, we identified an NKL-code in B-cell development whose violation may deregulate differentiation and promote malignant transformation.

## Introduction

Hematopoietic stem cells (HSCs) generate common myeloid and lymphoid progenitor (CMP/CLP) cells which, respectively, initiate the differentiation into myeloid and lymphoid cell lineages. The latter produces all types of lymphocytes comprising B-cells, T-cells, and NK-cells. Early B-cell development begins with the B-cell progenitor (BCP) and takes place in the bone marrow. The final differentiation steps to memory B-cells and plasma cells via naïve and germinal center (GC) B-cells occur in lymph nodes and in the spleen [1–3]. Lymphopoiesis including B-cell development is regulated mainly at the transcriptional level [3,4]. Accordingly, several transcription factors (TFs) including PAX5, MYB, BCL6 and PRDM1/BLIMP1 generate a B-cell specific regulatory network controlling fundamental differentiation processes [5,6]. Deregulation of these TFs by chromosomal rearrangement or gene mutation contributes to the generation of B-cell malignancies [7,8].

Homeobox genes encode TFs containing a homeodomain which mediates both sequence-specific DNA-binding and contacts to cofactors. Most of these TFs regulate basic developmental processes in both embryos and adults. The clustered homeobox genes are expressed within the embryo in a particular pattern termed the HOX-code. This code determines the anterior-posterior differentiation of the branchial and head region [9]. In the embryonic pharyngeal region DLX homeobox genes show a dorso-ventral expression pattern, termed DLX-code which provides a scaffold for the differentiation of tissues and organs in this body part [10]. Of note, both codes are generated by closely related homeobox gene groups showing high similarities in their encoded homeodomains.

Homeobox genes are arranged into classes and subclasses according to sequence differences of the conserved homeobox [11]. The human NKL homeobox gene subclass comprises 48 members and includes master genes controlling embryonal development of the heart (NKX2-5), prostate (NKX3-1) and spleen (NKX3-2 and TLX1) [12–14]. Recently, we analyzed the physiological expression of NKL homeobox genes in early hematopoiesis and T-cell development constituting a pattern termed NKL-code [15]. According to this code, HSCs express four different NKL homeobox genes (HHEX, HLX, NKX2-3, NKX3-1), CLPs three genes (HHEX, HLX, NANOG) and BCPs four genes (HHEX, HLX, MSX1, NKX6-3) while mature T-cells show absence of NKL homeobox gene activities. Thus, T-cell maturation requires silencing of all NKL subclass members.

To date, 24 aberrantly activated NKL homeobox genes have been described in T-cell acute lymphoid leukemia (T-ALL), representing the most prolific group of oncogenes in this malignancy [15,16]. These data imply that alterations of the NKL-code may underlie leukemogenesis by deregulation of differentiation processes. For example, NKL homeobox gene MSX1 is normally expressed in CLPs and remains active in NK-cells [17,18]. Accordingly, MSX1 represents an oncogene in T-ALL but a tumor suppressor gene in NK-cell leukemia [17,18]. Mechanisms of aberrant NKL homeobox gene activation in T-ALL include chromosomal rearrangements, altered chromatin structures, and deregulated TFs and signalling pathways [18–21].

Aberrant activities of NKL homeobox genes are reported in B-cell malignancies as well, comprising HLX in Hodgkin lymphoma (HL), MSX1 in mantle cell lymphoma (MCL), NKX2-1 in diffuse large B-cell lymphoma (DLBCL), and NKX2-3 in splenic marginal zone lymphoma (SMZL), mucosa-associated lymphoid tissue (MALT) lymphoma, and DLBCL [22–25]. However, no systematic analysis has been performed to evaluate the general role of this oncogene group in B-lymphoid cancers. Here, we extended our findings concerning physiological expression patterns of NKL homeobox genes in lymphopoiesis. We analyzed their activity in normal B-cell development and thereby broadened the applicability of the NKL-code to include this lymphocyte group. Subsequent screening of NKL homeobox gene expression in B-cell malignancies revealed both upregulated activities of NKL-code members and ectopic expression of non-hematopoietic genes. In addition, we performed detailed analyses of B-cell specific NKL homeobox gene NKX6-3 showing aberrant overexpression in subsets of DLBCL patients and cell lines.

## Materials and methods

### Expression profiling

Public expression profiling datasets used in this study were generated by U133 Plus 2.0 gene chips from Affymetrix and obtained from Gene Expression Omnibus (GEO, www.ncbi.nlm.nih.gov). Of note, this type of profiling gene chip contains 37 probes of 48 known human NKL homeobox genes. We exploited datasets GSE56315 and GSE12366 for analyses of developing B-cells [26,27], datasets GSE12453, GSE53786, GSE16455 and GSE21452 for that of B-cell lymphoma patient samples [28–31], and GSE42203 for B-cell lymphoma cell lines containing 39 DLBCL and 3 SMZL cell lines. Expression profiling data of selected B-cell lymphoma cell lines were obtained from the Cancer Cell Line Encyclopedia (CCLE) project (www.broadinstitute.org/ccle) and from Astra Zeneca (GSE57083). For analysis of NKX6-3 expression in cell lines these data were transformed as follows: after RMA-background correction and quantile normalization of the spot intensities, profiling data were expressed as ratios of the sample mean and subsequently log2 transformed. Data processing was performed via R/Bioconductor using Limma and Affy packages.

The dataset GSE69239 provides RNA sequencing data of several isolated hematopoietic entities [32]. The samples have been renamed, Thy1 (CD34+ CD7- CD1a-) into DN1, Thy2 (CD34+ CD7+ CD1a-) into DN2, Thy3 (CD34+ CD7+ CD1a+) into DN3, Thy4 (CD4+ CD8+) into DP, Thy5 (CD4+ CD8-) into SP4, and Thy6 (CD4- CD8+) into SP8. The expression data are given in units of FPKM (fragments per kilobase of mappable gene length and million reads) and were analyzed and interpreted as described previously [15].

### Cell lines and treatments

DLBCL cell lines, HL cell line L-540, and NIH-3T3 cells were obtained from the DSMZ (Braunschweig, Germany) and cultivated as described elsewhere [33]. To modify gene expression levels we used gene specific siRNA oligonucleotides in comparison to AllStars negative Control siRNA (siControl) which were obtained from Qiagen (Hilden, Germany). Gene expression constructs for HHEX, HLX, MSX1 and PCGF5 were cloned in vector pCMV6-XL4 and obtained from Origene (Wiesbaden, Germany). SiRNAs (80 pmol) and expression constructs/vector controls (2 μg) were transfected into 1x10^6^ cells by electroporation using the EPI-2500 impulse generator (Fischer, Heidelberg, Germany) at 350 V for 10 ms. Electroporated cells were harvested after 20 h cultivation. Cells were treated for 16 h with 20 ng/ml recombinant BMP7 (R & D Systems, Abingdon, UK), with BMP receptor inhibitor dorsomorphin (DM) (Calbiochem, Darmstadt, Germany) dissolved in dimethyl sulfoxide (DMSO) at a final concentration of 5 μM.

### Real-time quantitative polymerase chain-reaction (RQ-PCR) analyses

Total RNA was extracted from cultivated cell lines using TRIzol reagent (Invitrogen, Darmstadt, Germany). Primary human total RNA was commercially obtained. We used RNA from peripheral blood mononuclear cells (PBMC), thymus, lymph node (LN), spleen, and bone marrow (BM) obtained from Biochain/BioCat (Heidelberg, Germany), and RNA from peripheral CD19-positive B-cells and CD3-positive T-cells obtained from Miltenyi Biotec (Bergisch Gladbach, Germany). cDNA was synthesized using 5 μg RNA, random priming and Superscript II (Invitrogen). RQ-PCR analysis was performed using the 7500 Real-time System and commercial buffer and primer sets (Applied Biosystems/Life Technologies, Darmstadt, Germany). Quantification of MSX1 was performed as described recently [18]. For normalization of expression levels we quantified the transcripts of TATA box binding protein (TBP). Quantitative analyses were performed twice in triplicate. Standard deviations are presented in the figures as error bars. The statistical significance was assessed by Student´s T-Test and the calculated p-values were indicated by asterisks (* p<0.05, ** p<0.01, *** p<0.001, n.s. not significant).

### Protein analyses

To examine NKX6-3 protein expression we used primary antibody anti-NKX6-3 (Novus Biologicals, Abingdon, UK). Immuno-cytology was performed as follows: cells were spun onto slides and subsequently air-dried and fixed with methanol/acetic acid for 90 s. The antibody was diluted 1:20 in PBS containing 5% BSA and incubated for 30 min. Washing was performed 3 times with PBS. Preparations were incubated with secondary antibody (diluted 1:100) for 20 min. After final washing the cells were mounted in Vectashield (Vector Laboratories, Burlingame, CA) containing DAPI for nuclear staining. Documentation of subcellular protein localization was performed using an Axio-Imager microscope (Zeiss, Göttingen, Germany) configured to a dual Spectral Imaging FISH system (Applied Spectral Imaging, Neckarhausen, Germany).

Western blots were generated by the semi-dry method. Protein lysates from cell lines were prepared using SIGMAFast protease inhibitor cocktail (Sigma, Taufkirchen, Germany). Proteins were transferred onto nitrocellulose membranes (Bio-Rad, München, Germany) and blocked with 5% dry milk powder dissolved in phosphate-buffered-saline buffer (PBS). The following antibodies were used: alpha-Tubulin (Sigma), and SMAD1 (Santa Cruz Biotechnology, Heidelberg, Germany). For loading control blots were reversibly stained with Poinceau (Sigma) and detection of alpha-Tubulin (TUBA) was performed thereafter. Secondary antibodies were linked to peroxidase for detection by Western-Lightning-ECL (Perkin Elmer, Waltham, MA, USA). Documentation was performed using the digital system ChemoStar Imager (INTAS, Göttingen, Germany).

### Genomic and chromosomal analyses

For genomic profiling genomic DNA of DLBCL cell lines was prepared by the Qiagen Gentra Puregene Kit (Qiagen). Labelling, hybridization and scanning of Cytoscan HD arrays was performed at the Genome Analytics Facility, Helmholtz Centre for Infection Research (Braunschweig, Germany), according to the manufacturer´s protocols (Affymetrix, High Wycombe, UK). Data were interpreted using the Chromosome Analysis Suite software version 2.0.1.2 (Affymetrix).

Chromosomal analysis by FISH was performed as described previously [34]. BAC clones were obtained from BacPac Resources, Children´s Hospital Oakland Research Institute (CA, USA) to analyze the locus of NKX6-3 (RP11-45I11, RP11-141M18, RP11-265K5). BAC DNA was harvested using the Big BAC DNA Kit (Princeton Separations, Adelphia, NJ, USA) and directly labelled by nick translation with dUTP-fluors (Dyomics, Jena, Germany). Whole chromosome 8 painting probe was obtained from Applied Spectral Imaging (Neckarhausen, Germany). Fluorescent images were captured and analyzed with an Axio-Imager microscope (Zeiss) configured to a dual Spectral Imaging FISH system (Applied Spectral Imaging).

### Reporter gene assay

For creation of reporter gene constructs we combined a reporter with two different regulatory genomic fragments derived from the upstream region of NKX6-3, containing consensus binding sites for PAX5 and BACH2 and a binding site for MSX1 and HLX, respectively. The PAX5 site was visualized at the UCSC Genome Browser, the consensus binding sites for MSX1 and HLX are published elsewhere [35]. We cloned the genomic PCR products of the corresponding upstream region (regulator) and of the HOXA9 gene, comprising exon1-intron1-exon2 (reporter), into the *Hind*III/*Bam*HI and *Eco*RI sites, respectively, of the expression vector pcDNA3 downstream of the CMV enhancer. The oligonucleotides used for the amplification of the PAX5-site were obtained from Eurofins MWG (Ebersbach, Germany). Their sequences were as follows: NKX6-3-for1 5´-AAAAGCTTCCTGATCCTTCCTGGAGCAGG-3´, NKX6-3-rev1 5´-CTGGATCCTTTTACAGCAGAGAAAACTGAC-3´. The oligonucleotides used for the amplification of the MSX1/HLX-site were obtained from Eurofins MWG. Their sequences were as follows: NKX6-3-for2 5´-GCAAGCTTGGCTGCACGCTCCGGCTGCTAG-3´, NKX6-3-rev2 5´-TGGGATCCGAATGGTCTATACATCAGAGTAGG-3´. Introduced restriction sites used for cloning are underlined. Transfections of plasmid-DNA into NIH-3T3 cells were performed using SuperFect Transfection Reagent (Qiagen). Commercial HOXA9 and TBP assays were used for RQ-PCR to quantify the spliced reporter-transcript, corresponding to the regulator activity.

## Results

### NKL homeobox gene expression in normal B-cell development

**Fig 1:**
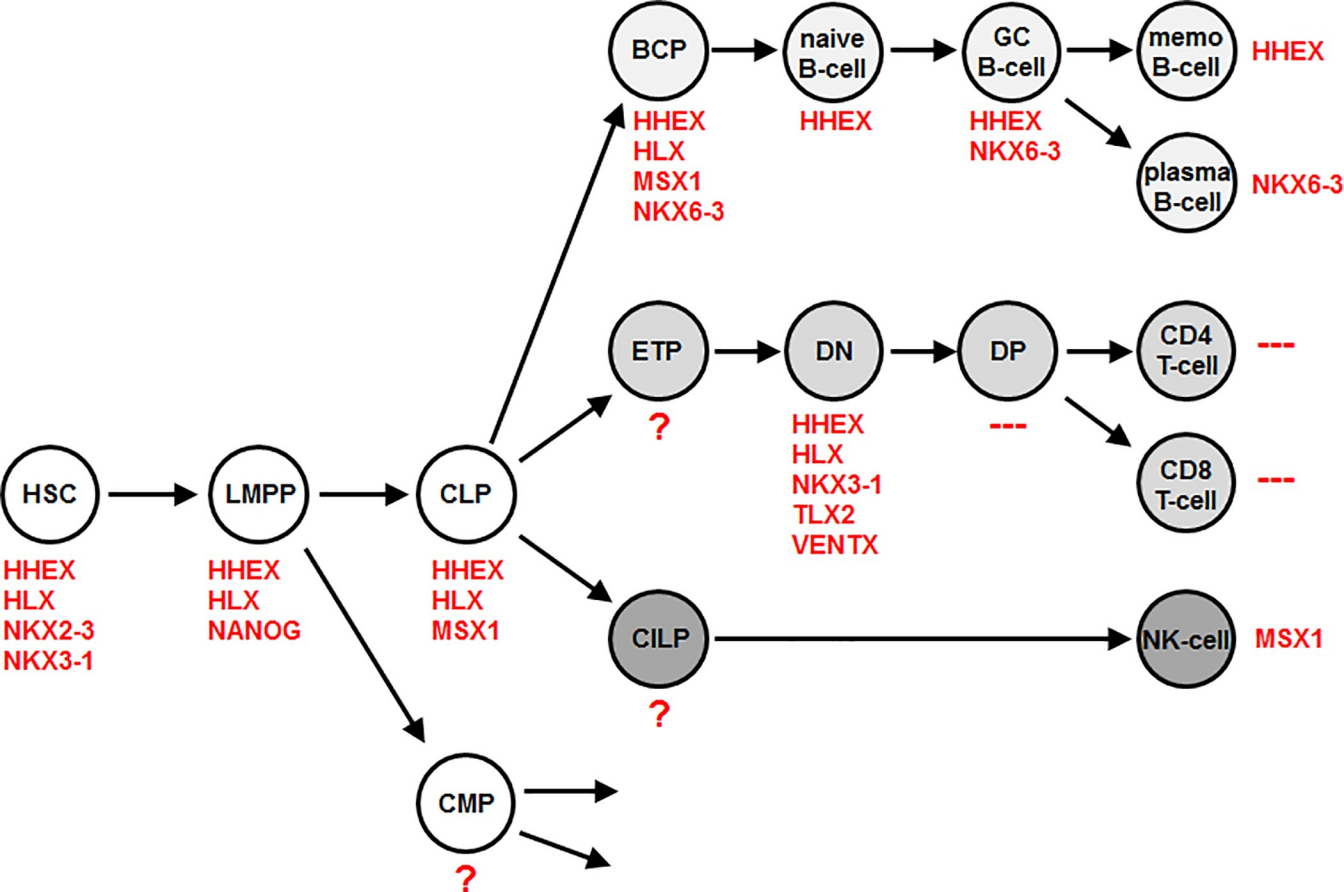
NKL-code in early hematopoiesis and lymphopoiesis. This figure summarizes the identified activities of NKL homeobox genes in particular hematopoietic developmental stages. The expression data were extracted from datasets GSE69239, GSE56315 and GSE12366 and published previously [15]. Abbreviations: hematopoietic stem cells (HSC), lymphoid and myeloid progenitor (LMPP), common lymphoid progenitor (CLP), common myeloid progenitor (CMP), B-cell progenitor (BCP), early T-cell progenitor (ETP), double negative T-cell (DN), double positive T-cell (DP), common innate lymphoid progenitor (CILP). The following acronyms were used for unknown gene activities (?), and absence of NKL homeobox gene expressions (—) in the indicated cell types.

Recently, we identified specific expression patterns of NKL homeobox genes in early hematopoiesis, lymphoid progenitors and T-cell development [15]. These data included samples of BCPs which expressed HHEX, HLX, MSX1 and NKX6-3 (**S1 Fig**). To analyze physiological expression patterns of NKL homeobox genes in subsequent stages of B-cell development we here exploited two public expression profiling datasets, GSE56315 and GSE12366, each comprising samples of naïve, germinal center (GC), memory B-cells, and plasma cells. From the NKL homeobox genes analyzed, corresponding activities at significant levels were detected for only HHEX and NKX6-3 in particular developmental stages of B-cells (**S2 Fig**). The results of this screening are shown in **Table 1** and **Fig 1**. Accordingly, naïve B-cells, GC B-cells, and memory B-cells expressed significant levels of HHEX. GC B-cells expressed NKX6-3 as well while plasma cells expressed NKX6-3 but not HHEX. Thus, these data extended the knowledge of the physiological activity of NKL homeobox genes during lymphopoiesis, showing an alternative NKL-code for the B-cell lineage. In contrast to mature T-cells, mature B-cells were positive for NKL homeobox gene activity, expressing either HHEX or NKX6-3.

**Table 1:** NKL homeobox gene expression in normal B-cell development and B-cell lymphomas.

Gene	HSC	LMPP	CLP	DN	BCP	NB	GCB	MB	PB	HL	FL	DLBCL	HCL	MCL	SMZL
**HHEX**	+	+	+	+	+	+	+	+					20		25
**HLX**	+	+	+	+	+					6	14			4	25
**MSX1**			+		+						20–28			3	
**NANOG**		+													
**NKX2-3**	+											2–9			
**NKX3-1**	+			+										4	
**NKX6-3**					+		+		+		14	2		4	
**TLX2**				+						6					
**VENTX**				+											
**BARX2**													20	14	
**DLX1**										6					
**EMX2**										6					
**NKX2-1**												7			
**NKX2-2**										12					
**NKX3-2**										6					

This table lists all NKL homeobox genes active in indicated entities of normal hematopoiesis and/or B-cell malignancy as identified in this and a previous study [15]. Positive expressions are displayed (+). Indicated values of the B-cell malignancies represent the percentages of positive cases per entity. The first nine NKL homeobox genes represent hematopoietic genes while the six below are non-hematopoietic. Abbreviations of normal hematopoietic stages: hematopoietic stem cells (HSC), lymphoid and myeloid progenitor (LMPP), common lymphoid progenitor (CLP), double negative thymocytes (DN), B-cell progenitor (BCP), naïve B-cells (NB), germinal center B-cells (GCB), memory B-cells (MB), plasma cells (PB).

### NKL homeobox gene expression in malignant B-cells

To identify aberrantly transcribed NKL homeobox genes in primary malignant B-cells we analyzed four expression profiling datasets including GSE12453 which contains 17 samples of HL, 4 T-cell rich B-cell lymphoma (TCRBL), 5 follicular lymphoma (FL), 5 Burkitt lymphoma (BL), and 11 DLBCL patients (**S3 Fig**). Dataset GSE53786 contains 119 samples of DLBCL patients (**S4 Fig**), and GSE16455 contains 22 samples of MCL, 5 hairy cell leukemia (HCL), 4 SMZL, 19 chronic lymphocytic leukemia (CLL), and 7 FL patients patients (**S5 Fig**). Finally, dataset GSE21452 contains 64 samples of MCL patients (**S6 Fig**). Seven of the nine expressed NKL homeobox genes in hematopoietic cells showed aberrant overexpression in subsets of B-cell malignancies, including HHEX, HLX, MSX1, NKX2-3, NKX3-1, NKX6-3 and TLX2 (**Table 1**). Furthermore, six NKL homeobox genes normally inactive in hematopoiesis were ectopically expressed. Accordingly, in HL patients we identified DLX1, EMX2, NKX2-2 and NKX3-2, in DLBCL we found NKX2-1, and HCL patients expressed BARX2 (**Table 1**). Some of these identified genes have been previously shown to be deregulated in subsets of particular B-cell lymphomas, including HLX in HL, MSX1 in MCL, NKX2-1 in DLBCL, and NKX2-3 in SMZL, MALT lymphoma and DLBCL [22–25]. Collectively, our data confirmed those published and revealed novel overexpressed subclass members in specific entity subsets. Furthermore, our data indicated that the role of NKL homeobox gene deregulation has been underestimated in B-lymphoid malignancies hitherto.

**Fig 2:**
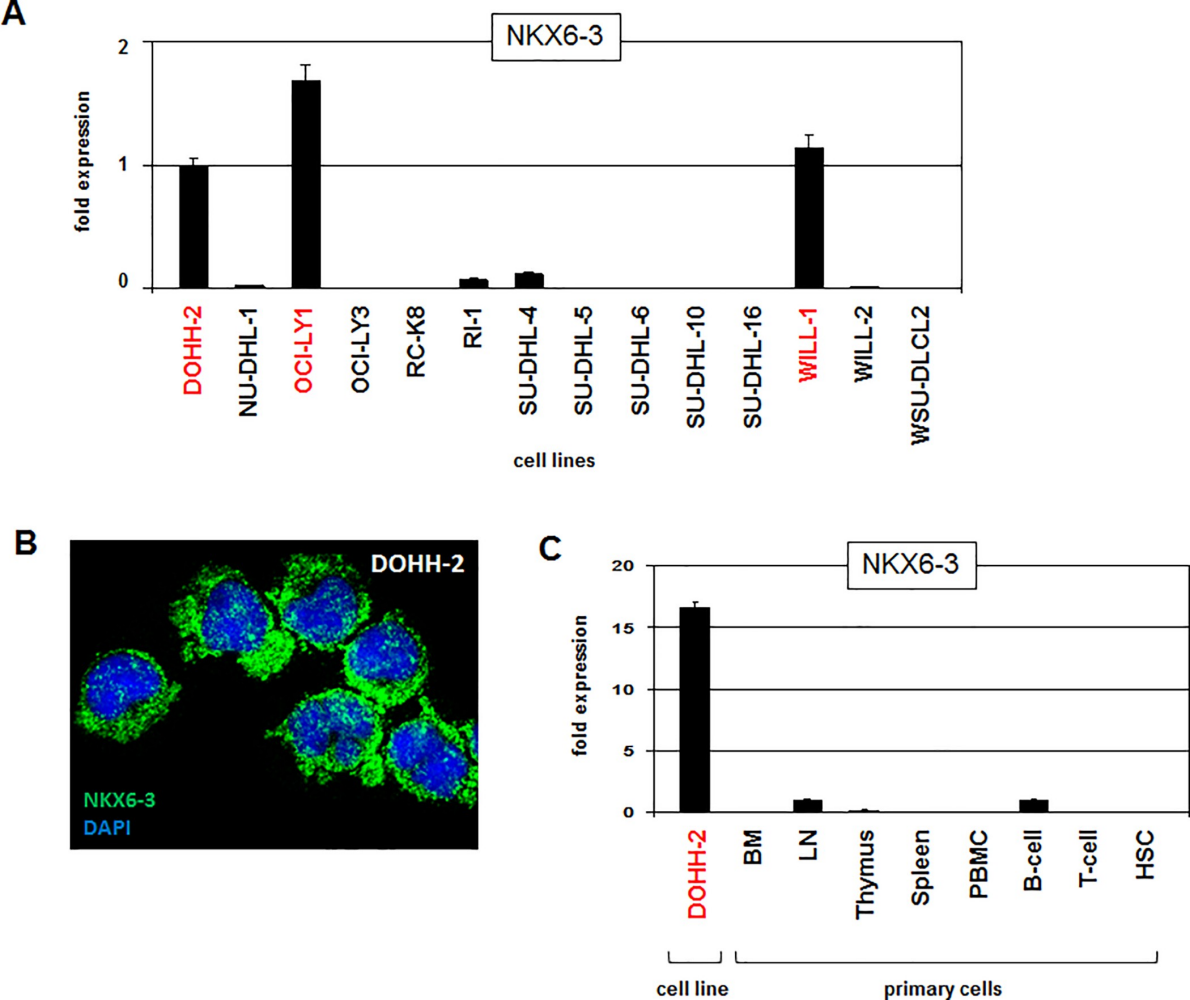
NKX6-3 expression in DLBCL cell lines. (A) RQ-PCR analysis of 14 DLBCL cell lines for NKX6-3. DOHH-2, OCI-LY1 and WILL-1 (indicated in red) demonstrated enhanced expression levels. (B) Immunocytological analysis of NKX6-3 protein (green signals) in DOHH-2 cells. The nucleus is counterstained with DAPI (blue). (C) RQ-PCR analysis of NKX6-3 in DOHH-2 and several primary hematopoietic cell/tissue samples including bone marrow (BM), lymph node (LN), thymus, spleen, peripheral blood mononuclear cells (PBMC), T-cells, B-cells, and hematopoietic stem cells (HSC).

Clinical data indicated that 14% of FL, 4% of MCL, and 2% of DLBCL patients showed aberrant overexpression of NKX6-3 (**Table 1**), indicating considerable deregulation in B-NHLs. Therefore, we focused on deregulated activities of this B-cell specific NKL homeobox gene. To identify cell line models for NKX6-3 we screened one FL cell line, four MCL cell lines and 14 DLBCL cell lines by expression profiling (**S1 Table**). This procedure revealed one DLBCL cell line (DOHH-2) which significantly overexpressed NKX6-3. Subsequent expression analysis of a similar set of 14 DLBCL cell lines via RQ-PCR confirmed enhanced NKX6-3 transcription in DOHH-2 and additionally in OCI-LY1 and WILL-1 as well (**Fig 2A**). In these three cell lines NKX6-3 expression was further demonstrated at the protein level by immunocytology. The signals were located both in the nucleus and in the cytoplasm as shown for DOHH-2 (**Fig 2B**), indicating functional relevance of NKX6-3 expression. RQ-PCR analysis of NKX6-3 in primary hematopoietic cell/tissue samples and DOHH-2 demonstrated expression in lymph nodes and mature B-cells. In contrast, the expression in bone marrow, thymus, spleen, peripheral blood cells, T-cells, and hematopoietic stem cells was nearly undetectable (**Fig 2C**). These results confirmed NKX6-3 overexpression in DLBCL cell line DOHH-2 which together with OCI-LY1 and WILL-1 represent, therefore, appropriate models to analyze upstream factors and downstream effects of NKX6-3 expression in B-cells.

### (De)regulation of NKX6-3 expression in DLBCL

**Fig 3:**
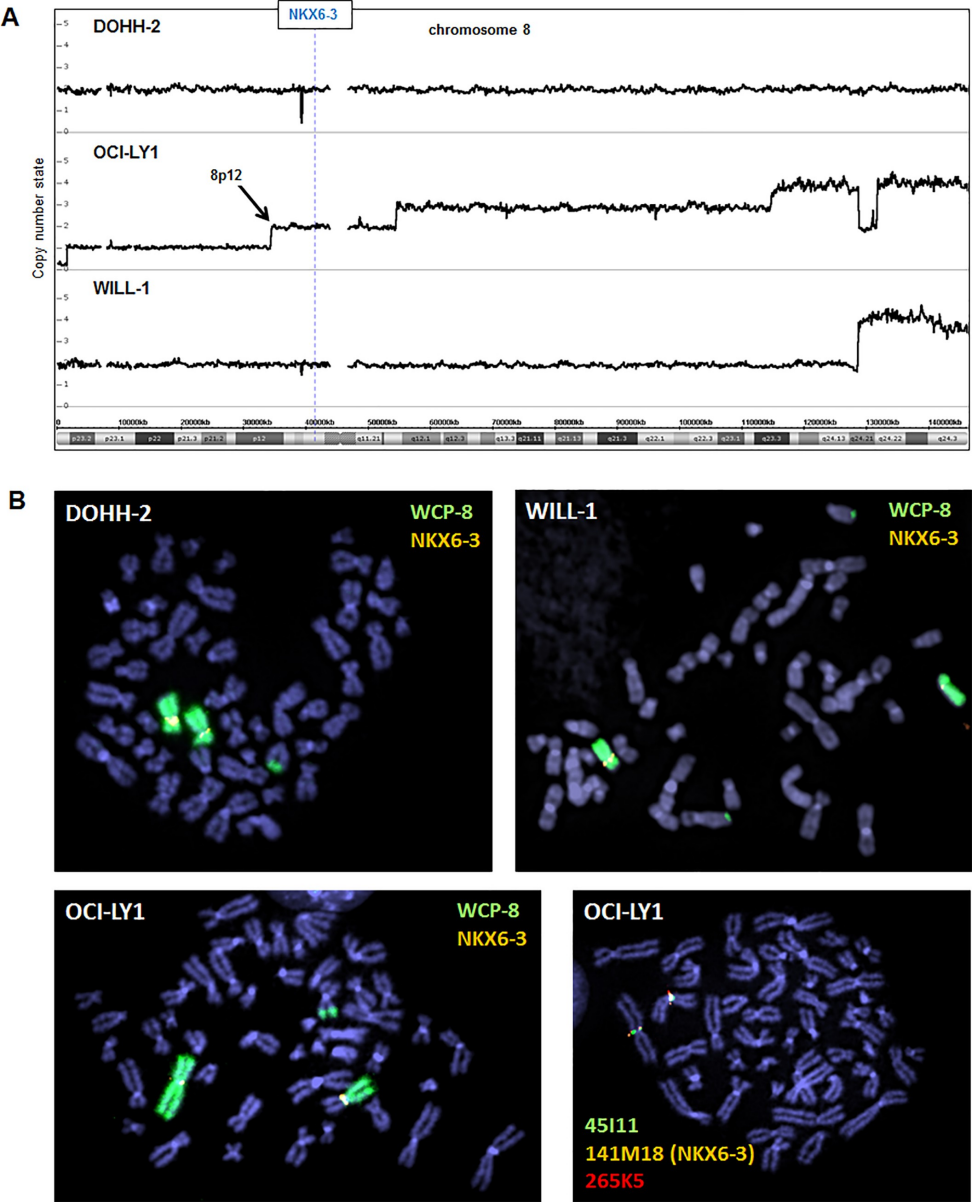
Genomic and chromosomal analysis of the NKX6-3 locus. (A) Genomic profiling data of DOHH-2, OCI-LY1 and WILL-1 allowed determination of the copy number state of all chromosomes including chromosome 8 shown here. The locus of NKX6-3 is indicated, demonstrating absence of aberrant alterations. OCI-LY1 contains at the region of 8p12 (arrow) a translocation breakpoint. (B) FISH analysis of NKX6-3 in DOHH-2, OCI-LY1 and WILL-1 was performed using whole chromosome paints (WCP) for chromosome 8 and BAC-probe 141M18 which covers the NKX6-3 gene. FISH analysis of OCI-LY1 using 141M18 in combination with flanking probes 45I11 and 265K5 excluded NKX6-3 as target gene of the indicated translocation.

In T-ALL several NKL homeobox genes are deregulated via chromosomal rearrangements [19]. To analyze if NKX6-3 overexpression was equally mediated via genomic aberrations we performed genomic profiling of DOHH-2, OCI-LY1 and WILL-1. While these cell lines contained several copy number alterations in their genomes, their NKX6-3 loci at chromosomal position 8p11 retained wild type configurations (**Fig 3A**). Furthermore, FISH analyses of DOHH-2, OCI-LY1 and WILL-1 excluded chromosomal translocations targeting the NKX6-3 gene (**Fig 3B**). Of note, OCI-LY1 contains a translocation at 8p12 but this breakpoint mapped 5 Mbp telomeric of NKX6-3 at UNC5D. Thus, these data excluded genomic rearrangements underlying enhanced NKX6-3 expression in DLBCL cell lines.

**Fig 4:**
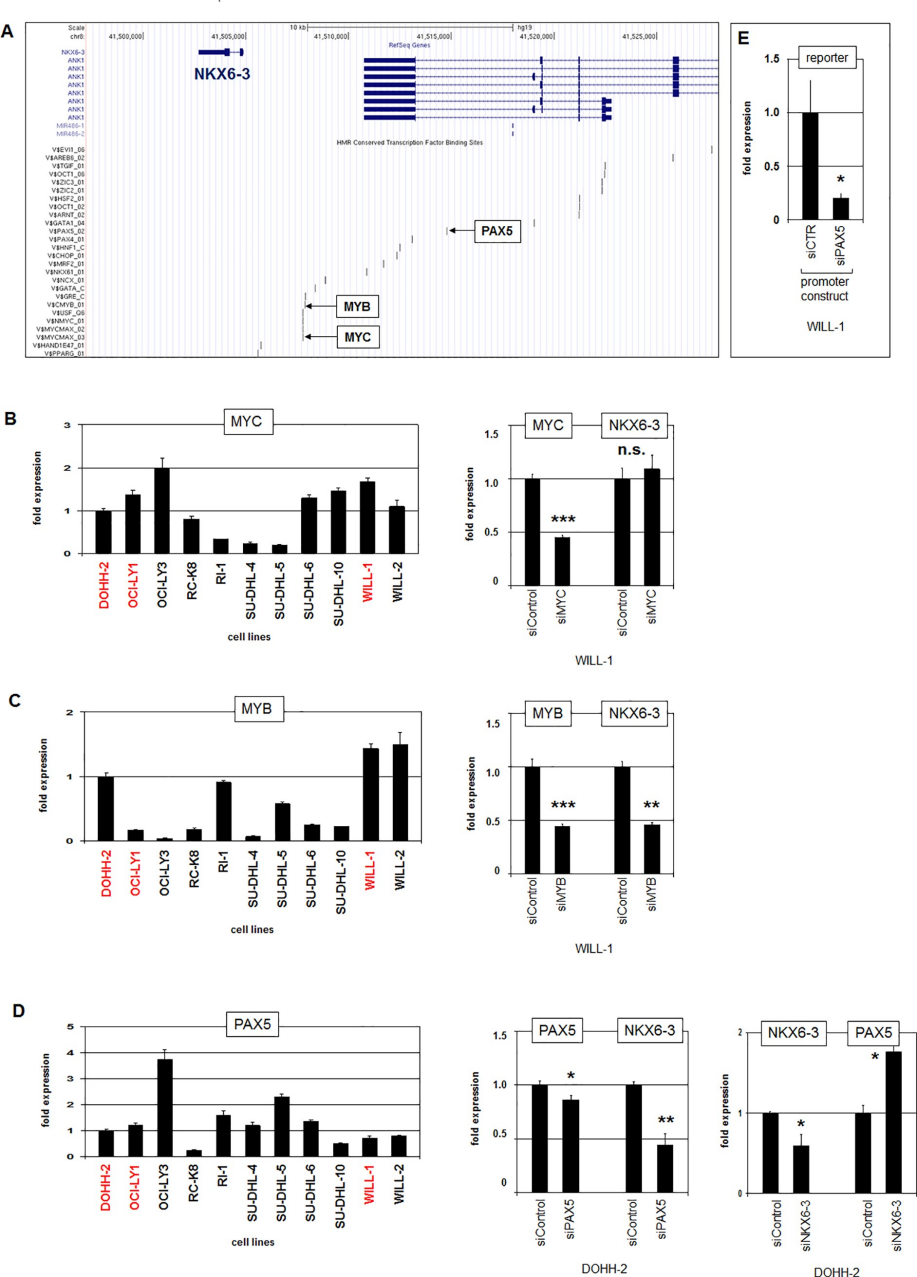
Activation of NKX6-3 transcription by MYB and PAX5. (A) According to the UCSC genome browser, the upstream region of NKX6-3 contains potential binding sites for several TFs including MYC, MYB and PAX5 as highlighted. (B) RQ-PCR analysis of 11 DLBCL cell lines for MYC demonstrating significant expression levels in DOHH-2, OCI-LY1 and WILL-1 (indicated in red, left). RQ-PCR analysis of WILL-1 cells treated for siRNA-mediated knockdown of MYC demonstrated reduced expression levels of MYC while the expression of NKX6-3 did not change significantly (right). (C) RQ-PCR analysis of 11 DLBCL cell lines for MYB demonstrating elevated expression levels in DOHH-2 and WILL-1 (left). RQ-PCR analysis of WILL-1 cells treated for siRNA-mediated knockdown of MYB demonstrated concomitantly reduced expression levels of MYB and NKX6-3 (right). (D) RQ-PCR analysis of 11 DLBCL cell lines for PAX5 demonstrating significant expression levels in DOHH-2, OCI-LY1 and WILL-1 (indicated in red, left). RQ-PCR analysis of DOHH-2 cells treated for siRNA-mediated knockdown of PAX5 demonstrated concomitantly reduced expression levels of PAX5 and NKX6-3 (middle). RQ-PCR analysis of DOHH-2 cells treated for siRNA-mediated knockdown of NKX6-3 demonstrated reduced expression levels of NKX6-3 and elevated levels of PAX5 indicating mutual regulation (right). (E) Reporter gene assay in WILL-1 cells using a construct which contained the indicated potential binding site for PAX5. In comparison to the control siRNA-mediated knockdown of PAX5 resulted in decreased expression levels of the reporter gene, indicating that PAX5 activates NKX6-3 transcription.

To identify candidate TFs involved in NKX6-3 (de)regulation we screened the promoter region of this NKL homeobox gene for consensus binding sites. The UCSC Genome Browser (www.genome.cse.ucsc.edu) revealed potential sites for the B-cell associated TFs MYC, MYB and PAX5 (**Fig 4A**). For these three factors/genes we performed RQ-PCR expression analyses and siRNA-mediated knockdowns in DLBCL cell lines (**Fig 4B–4D**). While the data for MYC showed no significant effect, those for MYB and PAX5 indicated an activating role. Furthermore, knockdown of NKX6-3 resulted in increased expression of PAX5 demonstrating mutual regulation of these genes. Finally, we performed a reporter-gene assay for the indicated PAX5-site (**Fig 4E**), confirming that the activating impact of PAX5 on NKX6-3 transcription operated directly. Thus, this promoter analysis demonstrated that NKX6-3 is activated by the TFs MYB and PAX5 –the latter represents a key master gene for B-cell development [36]. Therefore, mutual regulation of PAX5 and NKX6-3 via the feedback loop described above, define a new role for this NKL homeobox gene in B-cell differentiation processes.

To identify alternative mechanisms and factors involved in NKX6-3 activation we performed comparative expression profiling of DLBCL cell lines DOHH-2 and OCI-LY1 and six NKX6-3 negative controls using dataset GSE42203 (**S7 Fig**). The top 250 most differentially expressed genes included SMAD1 and BMP7—both components of the BMP-signalling-pathway [37]. Comparison of two NKX6-3 positive DLBCL patient samples with 10 negative controls using dataset GSE53786 confirmed significant overexpression of SMAD1 (**S8 Fig**). The gene encoding BMP7 lay out with the top 250 differentially expressed genes in this dataset but, nevertheless, BMP7 also showed statistically significant overexpression in NKX6-3 positive patients (**S8 Fig**). Furthermore, these patient data revealed significantly reduced expression levels of PCGF5 encoding a component of polycomb repressor complex (PRC)1.5 [38]. Thus, an enhanced BMP7/SMAD1-signalling pathway and reduced activity of PRC1.5 may contribute to NKX6-3 expression.

**Fig 5:**
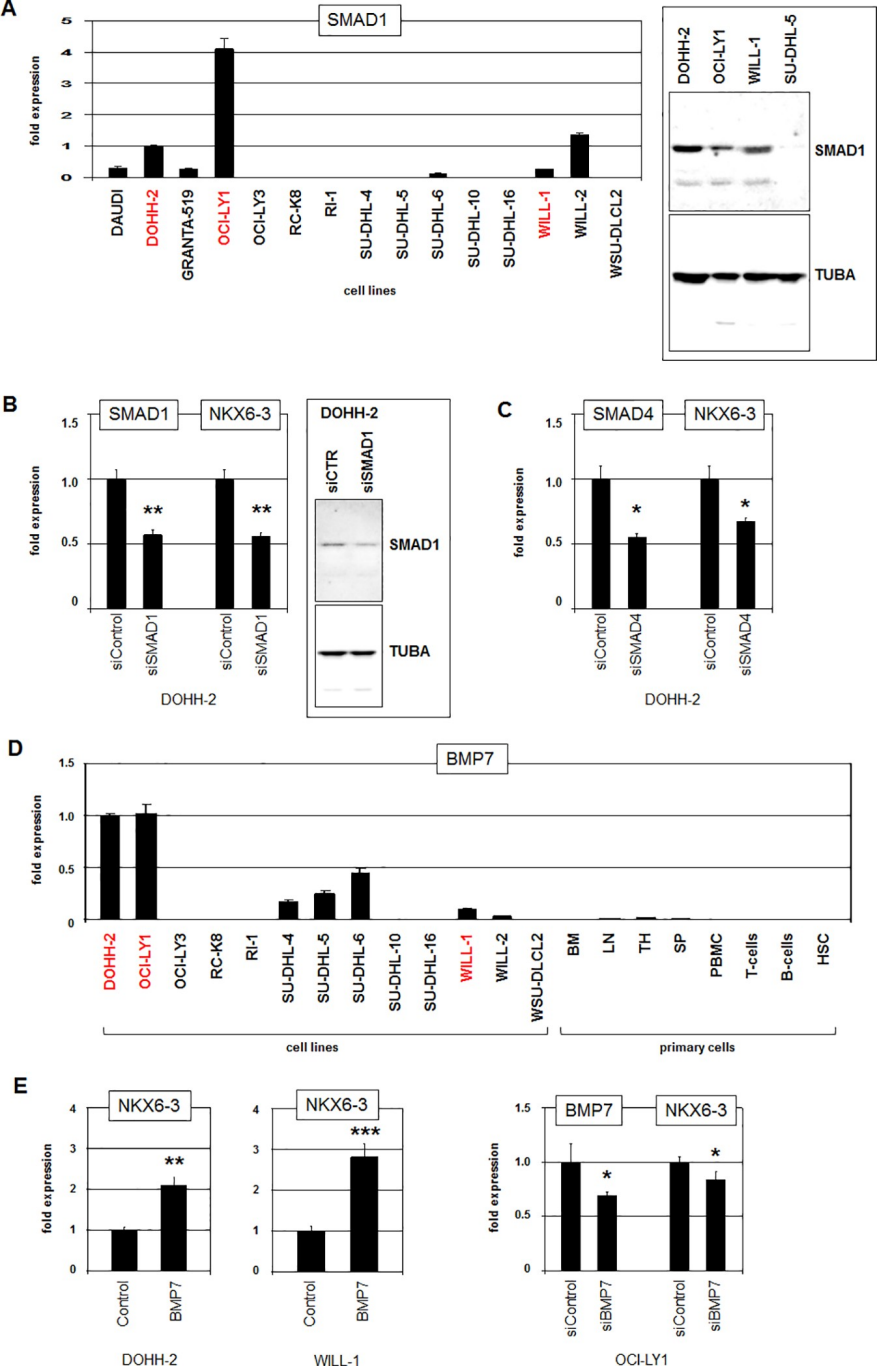
BMP-signalling pathway activates NKX6-3 expression. (A) RQ-PCR analysis of 13 DLBCL cell lines for SMAD1, demonstrating enhanced levels in DOHH-2 and OCI-LY1 (left). Westernblot analysis (right) confirmed SMAD1 expression at the protein level in DOHH-2, OCI-LY1 and WILL-1 while SU-DHL-5 served as negative control. Alpha-Tubulin (TUBA) served as loading control. (B) RQ-PCR analysis of SMAD1 and NKX6-3 after siRNA-mediated knockdown of SMAD1 in DOHH-2 cells demonstrated concomitantly reduced expression levels (left). SMAD1 knockdown was confirmed at the protein level by Western blot (right). (C) RQ-PCR analysis of SMAD4 and NKX6-3 after siRNA-mediated knockdown of SMAD4 in DOHH-2 cells demonstrated concomitantly reduced expression levels. (D) RQ-PCR analysis of 13 DLBCL cell lines and of primary hematopoietic cell/tissue samples for BMP7. DOHH-2, OCI-LY1 and WILL-1 (indicated in red) expressed significant levels of BMP7, contrasting the primary samples. (E) RQ-PCR analysis of NKX6-3 after treatment of DOHH-2 (left) and WILL-1 (middle) with recombinant BMP7 protein. RQ-PCR analysis of OCI-LY1 cells treated for siRNA-mediated knockdown of BMP7 demonstrated concomitantly reduced expression levels.

RQ-PCR and Western blot analyses of SMAD1 confirmed elevated expression levels in the NKX6-3 positive cell lines (**Fig 5A**). SiRNA-mediated knockdown of SMAD1 in DOHH-2 resulted in decreased expression levels of NKX6-3 (**Fig 5B**), demonstrating that SMAD1 mediated NKX6-3 activation. SiRNA-mediated knockdown of SMAD1-cofactor SMAD4 resulted in decreased NKX6-3 expression as well (**Fig 5C**), supporting the activatory impact of this SMAD-tandem. RQ-PCR analysis of BMP7 confirmed elevated expression levels in NKX6-3 positive cell lines (**Fig 5D**). Furthermore, primary hematopoietic cell/tissue samples showed low/absent BMP7 expression indicating ectopic activation of this gene in selected DLBCL cell lines (**Fig 5D**). Treatment of DOHH-2 and WILL-1 with recombinant BMP7 protein enhanced NKX6-3 expression (**Fig 5E**), while siRNA-mediated knockdown of BMP7 in DOHH-2 resulted in reduced expression of NKX6-3 (**Fig 5E**). Thus, autocrine BMP7/SMAD1-signalling activated NKX6-3 in DLBCL.

**Fig 6:**
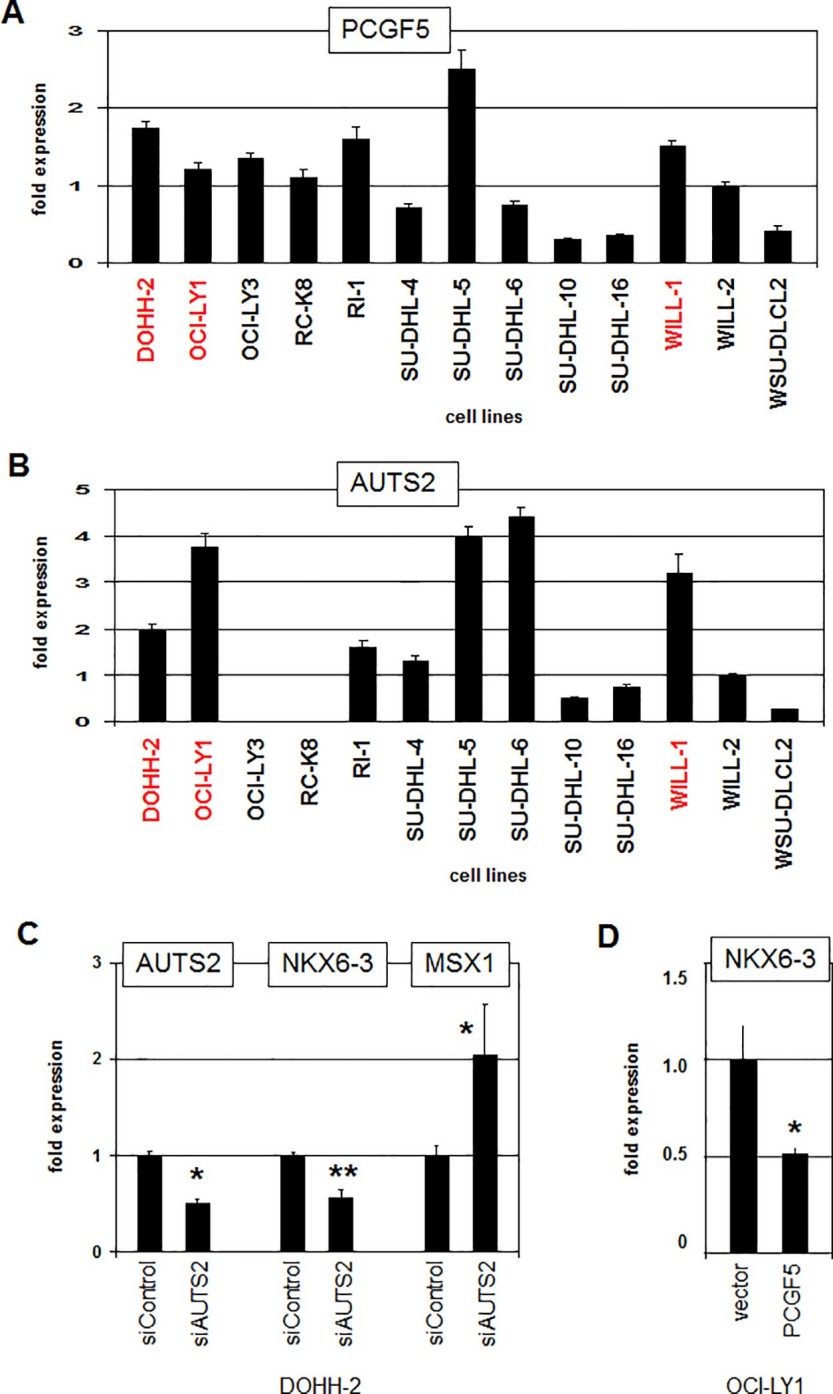
NKX6-3 regulation by AUTS2 and PCGF5. (A) RQ-PCR analysis of PCGF5 in 13 DLBCL cell lines demonstrated absence of reduced expression levels in DOHH-2, OCI-LY1 and WILL-1 (indicated in red). (B) RQ-PCR analysis of AUTS2 in 13 DLBCL cell lines indicated elevated expression levels in DOHH-2, OCI-LY1 and WILL-1. The indicated expression levels are related to WILL-2 (C) RQ-PCR analysis of DOHH-2 cells treated for siRNA-mediated knockdown of AUTS2 demonstrated concomitantly reduced expression levels of AUTS2 and NKX6-3 while the transcript levels of MSX1 increased. (D) RQ-PCR analysis of OCI-LY1 cells treated by forced expression of PCGF5 resulted in reduced transcript levels of NKX6-3.

PRCs are involved in the suppression of many homeobox genes notably clustered HOX genes [39]. However, RQ-PCR analysis of PRC1.5-component PCGF5 showed no significantly reduced expression levels in NKX6-3 positive cell lines (**Fig 6A**). In contrast RQ-PCR analysis of AUTS2 indicated elevated expression levels in these cell lines (**Fig 6B**). AUTS2 interacts with PCGF5 to convert this repressive factor into an activator [40]. This effect has been shown to play a role in aberrant activation of NKL homeobox gene MSX1 in T-ALL [18]. Here, siRNA-mediated knockdown of AUTS2 in DOHH-2 resulted in decreased expression of NKX6-3 (**Fig 6C**), indicating that NKX6-3 is activated by AUTS2 as well. Surprisingly, the expression level of MSX1 increased after AUTS2 knockdown (**Fig 6C**). This observation indicated mutual regulation of NKX6-3 and MSX1 (see below). Furthermore, forced expression of PCGF5 in OCI-LY1 resulted in reduced expression of NKX6-3 (**Fig 6D**). Thus, NKX6-3 transcription is regulated via the repressive chromatin complex PRC1.5 which is antagonized by AUTS2.

### Mutual regulation of B-cell associated NKL homeobox genes

**Fig 7:**
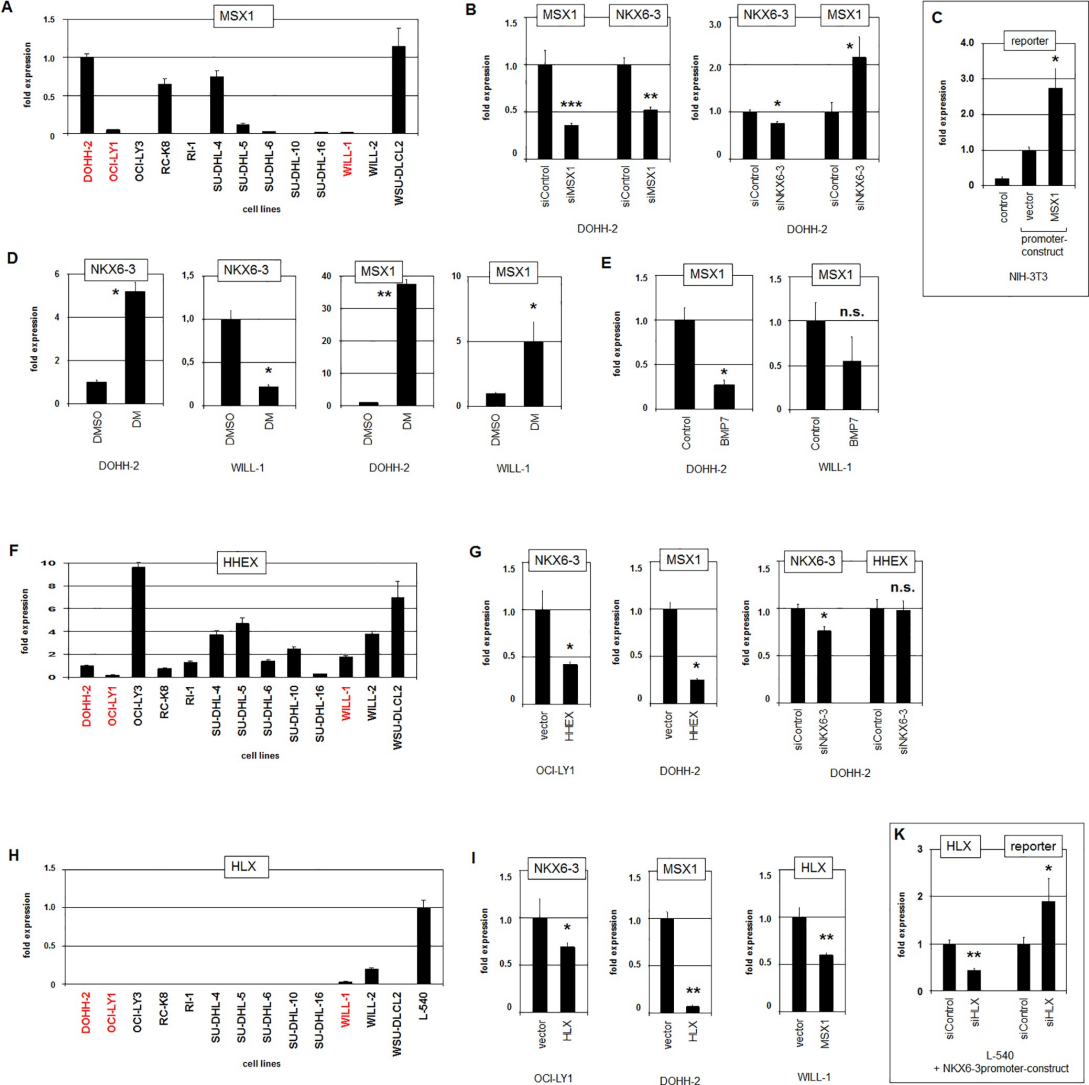
Mutual regulation of B-cell associated NKL homeobox genes. (A) RQ-PCR analysis of 13 DLBCL cell lines for MSX1 demonstrating elevated expression levels in DOHH-2 while OCI-LY1 and WILL-1 showed low MSX1 levels. (B) RQ-PCR analysis of DOHH-2 cells treated for siRNA-mediated knockdown of MSX1 demonstrated concomitantly reduced expression levels of MSX1 and NKX6-3 (left). RQ-PCR analysis of DOHH-2 cells treated for siRNA-mediated knockdown of NKX6-3 demonstrated reduced expression levels of NKX6-3 while the MSX1 transcript levels increased (right). (C) Reporter gene assay in NIH-3T3 cells using a construct which contained a potential binding site for MSX1 at position -6.011 bp of the NKX6-3 gene. In comparison to the vector control forced expression of MSX1 resulted in increased expression levels of the reporter gene, indicating that MSX1 activates NKX6-3 transcription. (D) RQ-PCR analysis for NKX6-3 of DOHH-2 and WILL-1 after treatment with BMP-receptor inhibitor dorsomorphin (DM) (left). RQ-PCR analysis for MSX1 of DOHH-2 and WILL-1 after treatment with DM (right). (E) RQ-PCR analysis for MSX1 of DOHH-2 (left) and WILL-1 (right) after treatment with recombinant BMP7 protein. (F) RQ-PCR analysis of 13 DLBCL cell lines for HHEX demonstrating reduced expression levels in DOHH-2, OCI-LY1 and WILL-1 (indicated in red). (G) RQ-PCR analysis for NKX6-3 of OCI-LY1 cells treated by forced expression with HHEX (left). RQ-PCR analysis for MSX1 of DOHH-2 cells treated by forced expression with HHEX (middle). RQ-PCR analysis of DOHH-2 cells treated by siRNA-mediated knockdown of NKX6-3 (right). While this treatment resulted in reduced expression levels of NKX6-3, no change in HHEX expression levels was detectable. (H) RQ-PCR analysis of 13 DLBCL cell lines and one HL control cell line (L-540) for HLX demonstrating reduced expression levels in all DLBCL cell lines. Of note, WILL-1 showed low transcript levels of HLX. (I) RQ-PCR analysis for NKX6-3 of OCI-LY1 treated by forced expression of HLX (left). RQ-PCR analysis for MSX1 of DOHH-2 treated by forced expression of HLX (middle). These data indicated that HLX repressed the expression of both NKX6-3 and MSX1. RQ-PCR analysis for HLX of WILL-1 treated by forced expression of MSX1 (right), indicating that MSX1 repressed the expression of HLX. (K) Reporter gene assay in NIH-3T3 cells using a construct which contained a potential binding site for HLX/MSX1 at position -6.011 bp of the NKX6-3 gene. In comparison to the control, siRNA-mediated knockdown of HLX resulted in increased expression levels of the reporter gene, indicating that HLX represses NKX6-3 transcription.

Our data obtained for AUTS2/PCGF5 indicated a potential regulatory role for NKX6-3 in MSX1 expression (**Fig 6C**). RQ-PCR analysis of MSX1 transcripts in DLBCL cell lines showed varying expression levels (**Fig 7A**). While DOHH-2 expressed high levels, OCI-LY1 and WILL-1 expressed very low MSX1 RNA levels. SiRNA-mediated knockdown of MSX1 in DOHH-2 resulted in reduced NKX6-3 expression while knockdown of NKX6-3 activated MSX1 expression (**Fig 7B**). Thus, MSX1 is an activator of NKX6-3 which in turn inhibits MSX1. To see if MSX1 regulates NKX6-3 directly, we screened the upstream-region of NKX6-3 for potential binding-sites. This exercise identified a consensus site for MSX1 at position –6.011 bp. Subsequently we performed a reporter-gene assay for the corresponding region (**Fig 7C**), demonstrating that MSX1 activated NKX6-3 transcription via this site.

Treatment of DOHH-2 and WILL-1 cells with BMP-receptor inhibitor dorsomorphin (DM) resulted in increased NKX6-3 levels in DOHH-2 but decreased levels in WILL-1 (**Fig 7D**). Furthermore, this treatment boosted MSX1 expression strongly in DOHH-2 but only moderately in WILL-1 (**Fig 7D**). Consistently, treatment of DOHH-2 and WILL-1 with BMP7 resulted in inhibition of MSX1 expression in both cell lines (**Fig 7E**). These data indicated that NKX6-3 and MSX1 were respectively activated and repressed by BMP7/SMAD1-signalling. However, the activating impact of MSX1 on NKX6-3 was only effective in DOHH-2 which expresses significant levels of both genes.

To see if in addition to MSX1 the remaining B-cell associated NKL homeobox genes HHEX and HLX also influence NKX6-3 expression, we examined their gene activities and impact. RQ-PCR analysis of HHEX demonstrated low transcript levels in NKX6-3 positive cell lines DOHH-2, OCI-LY1 and WILL-1 (**Fig 7F**). Accordingly, forced expression of HHEX in OCI-LY1 cells resulted in reduced NKX6-3 expression levels (**Fig 7G**), indicating that HHEX repressed NKX6-3 transcription. Forced expression of HHEX in DOHH-2 resulted in reduced transcription of MSX1 as well, indicating that HHEX repressed MSX1 in addition to NKX6-3 (**Fig 7G**). However, siRNA-mediated knockdown of NKX6-3 in DOHH-2 cells showed no significant alteration in HHEX expression levels (**Fig 7G**), discounting mutual regulation.

RQ-PCR analysis of HLX showed low/absent expression levels in all DLBCL cell lines examined (**Fig 7H**). As a control we additionally quantified HLX transcripts in HL cell line L-540 which has been shown to aberrantly express this NKL homeobox gene at a level similar to HSCs [22]. Forced expression of HLX in OCI-LY1 and DOHH-2 resulted in reduced transcription of NKX6-3 and MSX1, respectively, indicating inhibitory impacts (**Fig 7I**). Forced expression of MSX1 in WILL-1 resulted in reduced transcription of HLX, indicating mutual repression (**Fig 7I**). Finally, we reused the reporter-gene assay for HLX as described above for MSX1 because both factors bind the same consensus site. These data showed that HLX repressed NKX6-3 transcription directly (**Fig 7K**). Thus, MSX1 activated while HLX repressed NKX6-3 transcription, competing for the same binding site. Taken together, these data demonstrated that NKX6-3 is regulated by B-cell associated NKL homeobox genes MSX1, HHEX and HLX, forming a gene network.

## Discussion

**Fig 8:**
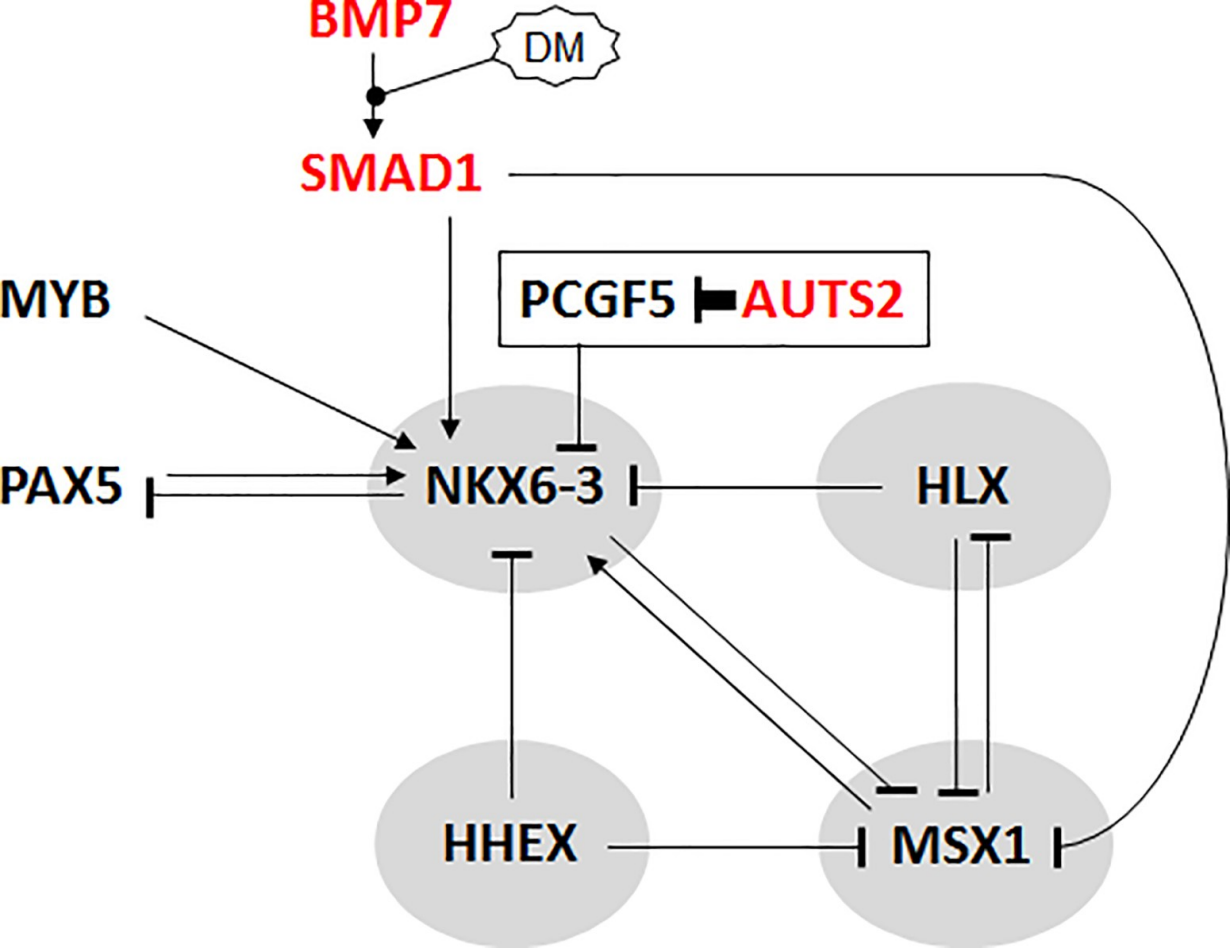
NKX6-3 is part of a gene regulatory network in B-cells. (A) This figure depicts a gene regulatory network showing NKX6-3 at a central position. B-cell associated NKL homeobox genes are indicated by a gray background, highly expressed activating factors in red letters.

In this study we analyzed physiological activities of NKL homeobox genes in B-cell development. Considered together with previously published data, this examination detected expression patterns which complete the NKL-code for early hematopoiesis and lymphopoiesis (**Fig 1**). Additional results of our work are summarized in **Fig 8**, which shows a gene regulatory network surrounding NKL homeobox gene NKX6-3. TFs MYB and PAX5, BMP7/SMAD1-signalling, and PRC1.5-mediator AUTS2 all activate expression of NKX6-3.

BCPs express four different NKL homeobox genes [15]. In subsequent stages of B-cell development only HHEX and NKX6-3 remain active while HLX and MSX1 are switched off. Memory B-cells and plasma cells represent the two final stages of B-cell differentiation expressing HHEX and NKX6-3, respectively. Thus, while mature NK-cells express MSX1 and mature B-cells HHEX or NKX6-3, mature T-cells lack any NKL homeobox gene activity [15,17]. Aberrant activation of NKL homeobox genes in T-cell progenitors arrests the T-cell differentiation program and promotes their malignant transformation [41–43]. In T-ALL, 24 aberrantly expressed NKL homeobox genes have been described to date, including seven hematopoietic and 17 non-hematopoietic genes [15,16]. In B-cell malignancies, we identified aberrant expression of seven hematopoietic and six non-hematopoietic NKL homeobox genes—a considerably lower count of deregulated subclass members. Notwithstanding this difference, these data suggested that NKL homeobox genes play a distinct role in the process of malignant transformation of B-cells as well.

NKL homeobox gene NKX6-3 is physiologically expressed in the course of B-cell development and aberrantly overexpressed in several B-cell malignancies including FL, MCL and DLBCL. Three NKX6-3 overexpressing cell lines derived from DLBCL were identified and used as models to reveal upstream and downstream factors of this candidate oncogene. First, we excluded genomic and chromosomal rearrangements possibly involved in NKX6-3 deregulation. Thus, while chromosomal rearrangements frequently mediate oncogenic activation of NKL homeobox genes in T-ALL [16,19], this type of activation seems to be less common in B-cell malignancies–an exception being SMZL where aberrant activation of NKX2-3 occurs by a chromosomal translocation, t(10;14)(q24;q32), which juxtaposes this NKL homeobox gene with the IGH locus [25].

Then, we identified TFs MYB and PAX5 as (physiological) upstream regulators of NKX6-3. While MYB represents a general hematopoietic TF PAX5 is a master factor for B-cell development [6]. Of note, in developing B-cells MYB and PAX5 regulate the expression of RAG2 and AICDA [44,45], highlighting their coordinated role in B-cell differentiation. In DLBCL aberrant activity of the BMP7/SMAD1-pathway was found to be involved in overexpression of NKX6-3. SMAD1 is physiologically highly expressed in CLPs and BCPs while BMP7 is silent in these progenitors (**S1 Fig**) and in other hematopoietic cells, suggesting aberrant reactivation of SMAD1 via ectopically expressed BMP7. Deregulated BMP-signalling has been shown to activate MSX1 in T-ALL [18], highlighting this pathway in aberrant expression of NKL homeobox genes in lymphoid malignancies.

Deregulation via AUTS2/PCFG5 represents another concordance in oncogenic activation between NKX6-3 in DLBCL and MSX1 in T-ALL [20]. AUTS2 interacts with polycomb repressor PCGF5 and recruits histone acetyltransferase EP300, relaxing chromatin conformation to promote gene activation [40]. Moreover, AUTS2 is also highly expressed in CLPs and BCPs (**S1 Fig**), supporting the idea of oncogenic reactivation of regulatory factors normally operating in progenitor cells. Chromatin complexes and modified histones are common regulators of homeobox genes [39,46]. Bivalent chromatin represents a special subtype and is formed by a unique combination of histone modifications at particular enhancer regions in embryonic stem and progenitor cells. Developmental genes including NKL homeobox genes are frequently regulated by this type of chromatin alteration [47]. Accordingly, aberrant modification of bivalent chromatin components has been correlated with deregulated activity of several NKL homeobox genes in cancer including B-cell lymphoma, highlighting the role of chromatin structure for the activity of these key developmental/oncogenic genes [48]. Therefore, deregulation of specific chromatin components impacts the expression of NKL homeobox genes in DLBCL as shown here for NKX6-3 and as reported previously for MSX1 and NKX2-1 [20,24].

Transcriptional analyses of all four B-cell associated NKL homeobox genes revealed mutual regulation. MSX1 repressed HLX but activated NKX6-3. Furthermore, HHEX and HLX acted as repressors of NKX6-3 and MSX1. In HL, aberrantly expressed HLX represses the transcription of MSX1 as well, supporting the importance of this regulatory connection in developing B-cells [22]. Due to identical DNA binding potentials, HLX and MSX1 may compete for the regulation of NKX6-3 transcription, indicating highly balanced interdependencies. While SMAD1 activated NKX6-3 expression, stimulation of the BMP/SMAD-pathway repressed MSX1 as shown here and reported previously [18]. These opposing effects provide additional controls for this regulatory network. Although here examined exclusively in DLBCL cell lines, these relationships may as well occur in normal progenitor cells. Therefore, NKL homeobox genes HHEX, HLX MSX1 and NKX6-3 generate a regulatory network which may underlie the observed expression pattern in normal B-cell development constituting the NKL-code.

In conclusion, we identified particular NKL homeobox gene activities in B-cell development, thus completing the NKL-code for lymphopoiesis. According to this code we were able to reveal aberrantly expressed NKL homeobox genes in B-cell malignancies. Although less frequent when compared to T-ALL, NKL homeobox gene deregulation plays a considerable role in B-cell cancers as well. NKX6-3 is physiologically expressed in B-cell differentiation and aberrantly overexpressed in B-cell lymphomas. These data may contribute to the understanding of normal and aberrant B-cell differentiation.

## Supporting information

S1 Fig Selected gene expression levels (GSE69239). RNA-seq data from samples of early hematopoiesis and of T-cell development (GSE69239) were used to analyze expression of particular genes including NKL homeobox genes and regulators of NKX6-3. Expression levels are indicated in FPKM (fragments per kilobase of mappable gene length and million reads). HSPC: hematopoietic stem and progenitor cells, LMPP: lymphoid and myeloid progenitor, CLP: common lymphoid progenitor, BCP: B-cell progenitor, DN: double negative T-cell progenitor, DP: double positive T-cell progenitor, SP4: single positive CD4+ T-cell progenitor, SP8: single positive CD8+ T-cell progenitor. The samples of CLPs and BCPs were of special interest and are highlighted in red.
(TIF)

S2 Fig Gene expression levels of HHEX and NKX6-3 during B-cell development (GSE56315 and GSE12366). Expression profiling datasets GSE56315 and GSE12366 contain samples from B-cell developmental stages including naïve B-cells, GC B-cells, memory B-cells and plasma cells. Significant positive expression were revealed by cutoffs defined at 6 (GSE56315) and at 200 (GSE12366). Using these criteria just two NKL homeobox genes, HHEX and NKX6-3, were identified to be consistently expressed in both datasets in any of these types of B-cell.
(TIF)

S3 Fig Gene expression levels of selected NKL homeobox genes in B-cell lymphomas (GSE12453). Expression profiling dataset GSE12453 contains patient samples for Hodgkin lymphoma (HL), classical HL (cHL), nodular lymphocyte-predominant HL (NLPHL), T-cell rich B-cell lymphoma (TCRBL), Burkitt lymphoma (BL), diffuse large B-cell lymphoma (DLBCL), in addition to samples from normal B-cells including naïve (N), memory (M), germinal center (GC) and plasma cells (P). Boxplots were performed for expression levels from indicated types of lymphomas in comparison to normal B-cells. Outliers were defined as aberrant overexpression and highlighted by red arrowheads. Accordingly, DLX1, EMX2, HLX, NKX3-2 and TLX2 were each overexpressed in 1/7 (6%) and NKX2-2 in 2/7 (12%) of HL patients. MSX1 was overexpressed in 1/5 (20%) of FL patients, NKX2-3 was overexpressed in 1/11 (9%) of DLBCL patients. The expression level for MSX1 in normal memory B-cells (sample GSM312686) was interpreted as outlier and excluded (X).
(TIF)

S4 Fig Gene expression levels of selected NKL homeobox genes in DLBCL (GSE53786). Expression profiling dataset GSE53786 contains patient samples from 119 diffuse large B-cell lymphoma (DLBCL). Boxplots were performed for expression levels and outliers were defined as aberrant overexpression, highlighted by red arrowheads. Accordingly, NKX2-1 was overexpressed in 8/119 (7%) of DLBCL patients, NKX2-3 in 3/119 (2%) of patients, and NKX6-3 in 2/119 (2%) of DLBCL patients.
(TIF)

S5 Fig Gene expression levels of selected NKL homeobox genes in B-cell lymphomas (GSE16455). Expression profiling dataset GSE16455 contains patient samples for mantle cell lymphoma (MCL), hairy cell leukemia (HCL), splenic marginal zone lymphoma (SMZL), chronic lymphocytic leukemia (CLL), and follicular lymphoma (FL). We set the cutoff at 100 for positive gene activity. Boxplots were performed for expression levels and outliers were defined as aberrant overexpression, highlighted by red arrowheads. Accordingly, BARX2 and HHEX were each overexpressed in 1/5 (20%) of HCL patients. BARX2 was overexpressed in 3/22 (14%) of MCL patients. HHEX and HLX were each overexpressed in 1/4 (25%) of SMZL patients. HLX, NKX3-1 and NKX6-3 were each overexpressed in 1/22 (4%) of MCL patients. HLX and NKX6-3 were each overexpressed in 1/7 (14%) and MSX1 in 2/7 (28%) of FL patients.
(TIF)

S6 Fig Gene expression levels of selected NKL homeobox genes in MCL (GSE21452). Expression profiling dataset GSE21452 contains 64 patient samples for mantle cell lymphoma. We defined a cutoff at 8 to reveal positive gene activity. Boxplots were performed for expression levels and outliers were defined as aberrant overexpression, highlighted by red arrowheads. Accordingly, MSX1 was overexpressed in 2/64 (3%) of MCL patients. Of note, in this dataset no overexpression of HLX and NKX3-1 was detectable–overexpression of NKX6-3 was not significant.
(TIF)

S7 Fig Comparative gene expression profiling in DLBCL cell lines (GSE42203). Expression profiling dataset GSE42203 contains two DLBCL cell line samples (DOHH-2 and OCI-LY1, blue background) showing overexpression of NKX6-3. Expression levels of NKX6-3, SMAD1 and BMP7 were shown in comparison to six control DLBCL cell lines (SU-DHL-16, SU-DHL-10, SU-DHL-8, SU-DHL-5, SU-DHL-4, SU-DHL-7, red background). The statistical significances are indicated as p-values.
(TIF)

S8 Fig Comparative gene expression profiling in DLBCL patient samples (GSE53786). Expression profiling dataset GSE53786 contains two DLBCL patient samples showing overexpression of NKX6-3 (blue background). Expression levels of NKX6-3, SMAD1, BMP7 and PCGF5 are shown in comparison to 10 control DLBCL patients (red background). The statistical significances are indicated as p-values.
(TIF)

S1 Table Expression levels of NKX6-3 in selected cell lines. Expression profiling data for NKX6-3 of 19 B-cell lines are indicated in addition to the source of data and the derived malignancy (left). Representation of the data in a boxplot demonstrates the value of DOHH-2 as outlier, demonstrating NKX6-3 overexpression in this cell line (right).
(TIF)
