# Supplementary figures and images for: NKL homeobox gene activities in B-cell development and lymphomas
Source: PLoS One. 2018 Oct 11;13(10):e0205537. doi: 10.1371/journal.pone.0205537 (PMC6181399; doi:10.1371/journal.pone.0205537)

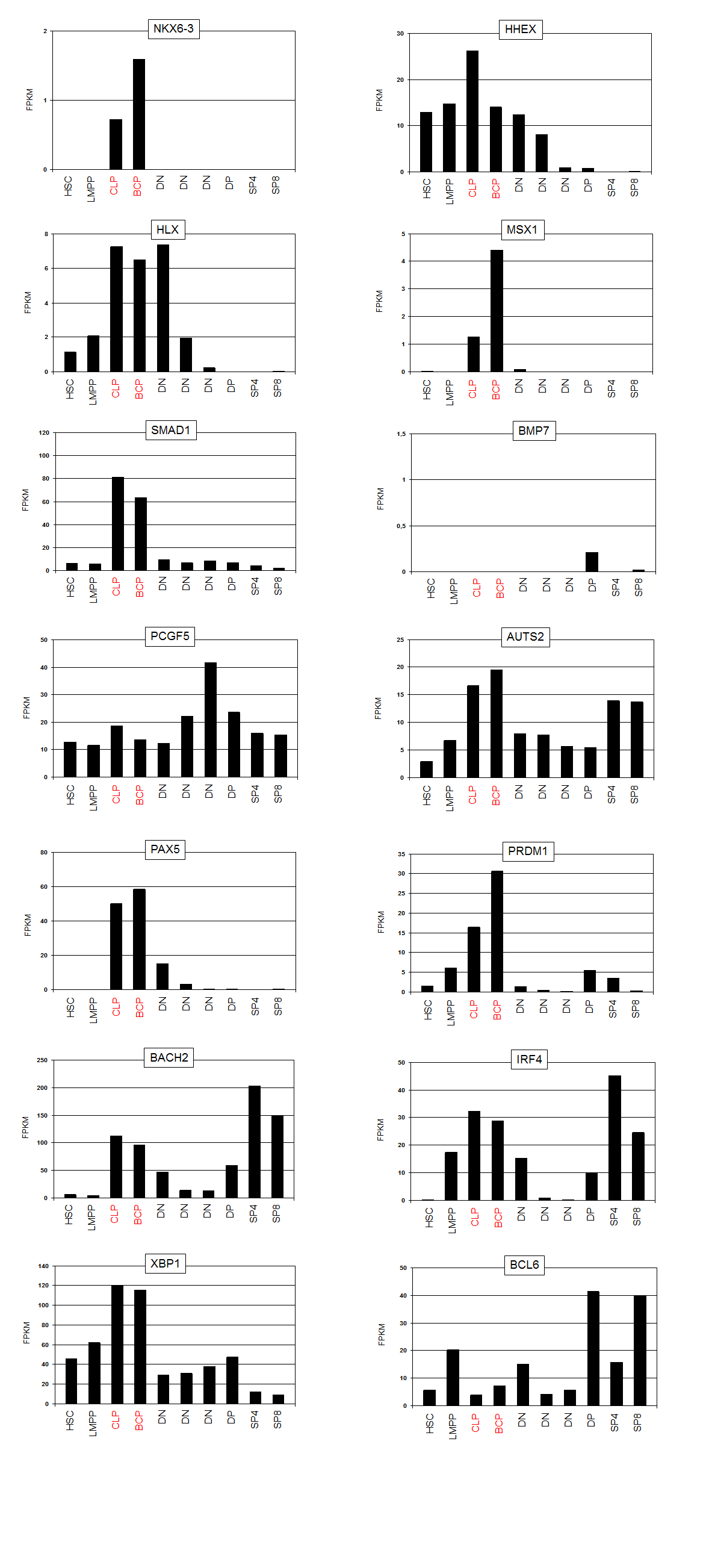

Supplement: S1 Fig — RNA-seq data from samples of early hematopoiesis and of T-cell development (GSE69239) were used to analyze expression of particular genes including NKL homeobox genes and regulators of NKX6-3. Expression levels are indicated in FPKM (fragments per kilobase of mappable gene length and million reads). HSPC: hematopoietic stem and progenitor cells, LMPP: lymphoid and myeloid progenitor, CLP: common lymphoid progenitor, BCP: B-cell progenitor, DN: double negative T-cell progenitor, DP: double positive T-cell progenitor, SP4: single positive CD4+ T-cell progenitor, SP8: single positive CD8+ T-cell progenitor. The samples of CLPs and BCPs were of special interest and are highlighted in red. (TIF) [file pone.0205537.s001.tif]

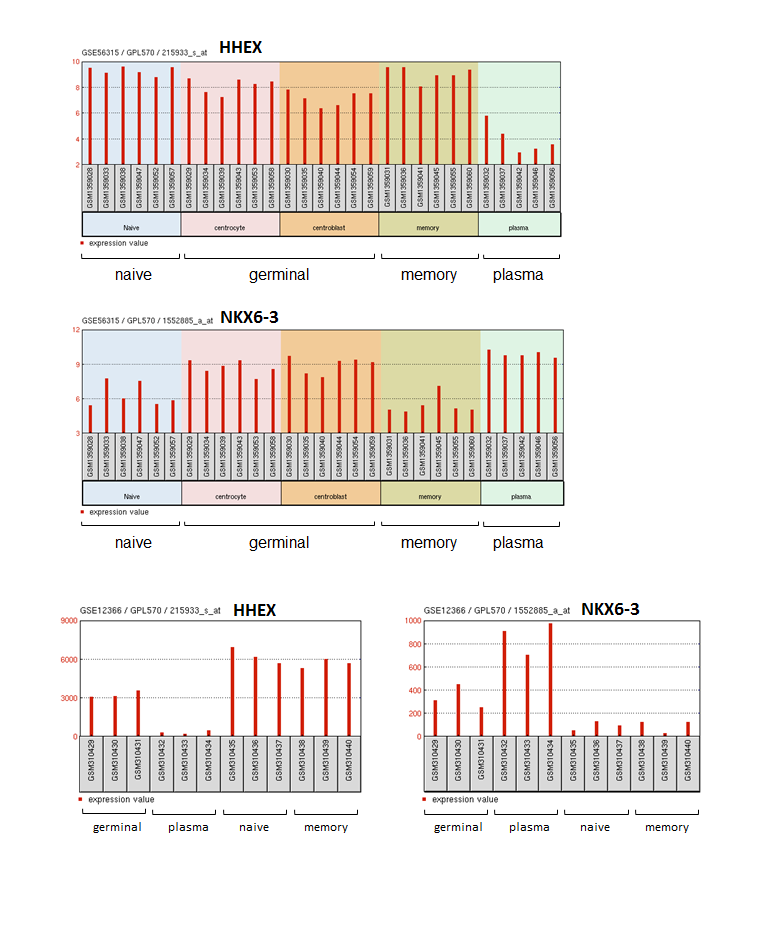

Supplement: S2 Fig — Expression profiling datasets GSE56315 and GSE12366 contain samples from B-cell developmental stages including naïve B-cells, GC B-cells, memory B-cells and plasma cells. Significant positive expression were revealed by cutoffs defined at 6 (GSE56315) and at 200 (GSE12366). Using these criteria just two NKL homeobox genes, HHEX and NKX6-3, were identified to be consistently expressed in both datasets in any of these types of B-cell. (TIF) [file pone.0205537.s002.tif]

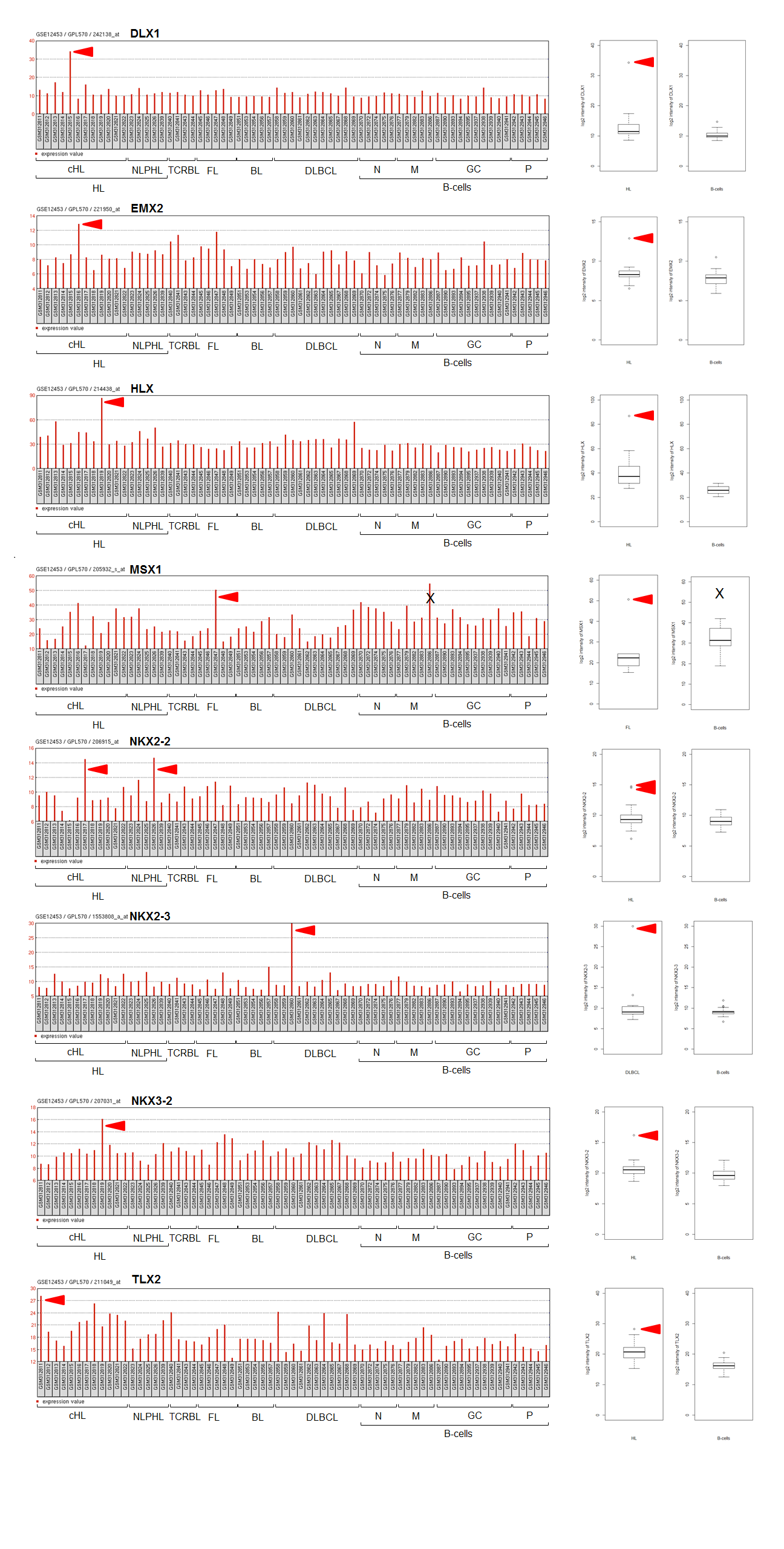

Supplement: S3 Fig — Expression profiling dataset GSE12453 contains patient samples for Hodgkin lymphoma (HL), classical HL (cHL), nodular lymphocyte-predominant HL (NLPHL), T-cell rich B-cell lymphoma (TCRBL), Burkitt lymphoma (BL), diffuse large B-cell lymphoma (DLBCL), in addition to samples from normal B-cells including naïve (N), memory (M), germinal center (GC) and plasma cells (P). Boxplots were performed for expression levels from indicated types of lymphomas in comparison to normal B-cells. Outliers were defined as aberrant overexpression and highlighted by red arrowheads. Accordingly, DLX1, EMX2, HLX, NKX3-2 and TLX2 were each overexpressed in 1/7 (6%) and NKX2-2 in 2/7 (12%) of HL patients. MSX1 was overexpressed in 1/5 (20%) of FL patients, NKX2-3 was overexpressed in 1/11 (9%) of DLBCL patients. The expression level for MSX1 in normal memory B-cells (sample GSM312686) was interpreted as outlier and excluded (X). (TIF) [file pone.0205537.s003.tif]

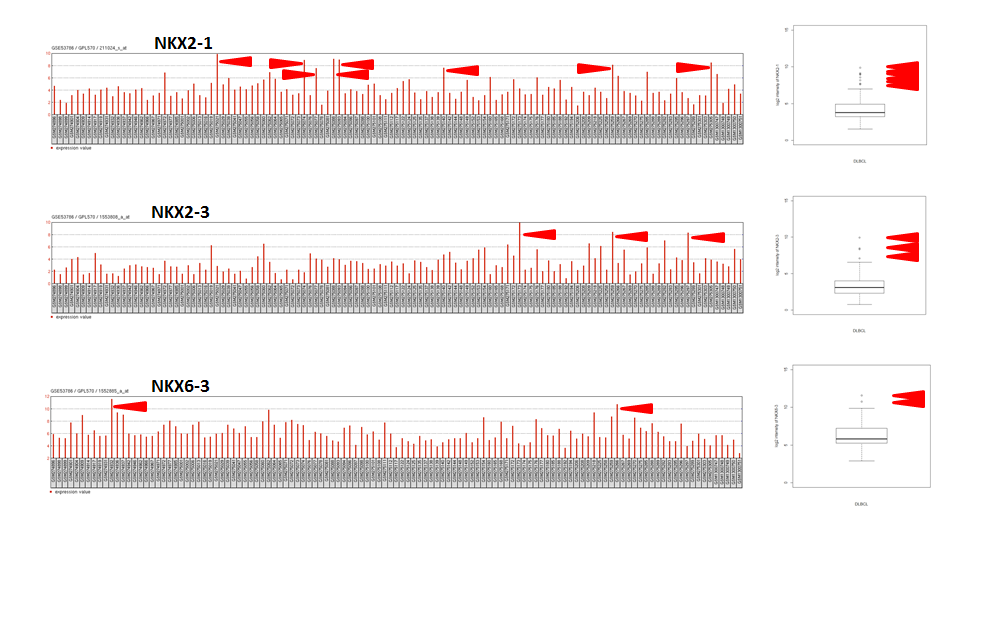

Supplement: S4 Fig — Expression profiling dataset GSE53786 contains patient samples from 119 diffuse large B-cell lymphoma (DLBCL). Boxplots were performed for expression levels and outliers were defined as aberrant overexpression, highlighted by red arrowheads. Accordingly, NKX2-1 was overexpressed in 8/119 (7%) of DLBCL patients, NKX2-3 in 3/119 (2%) of patients, and NKX6-3 in 2/119 (2%) of DLBCL patients. (TIF) [file pone.0205537.s004.tif]

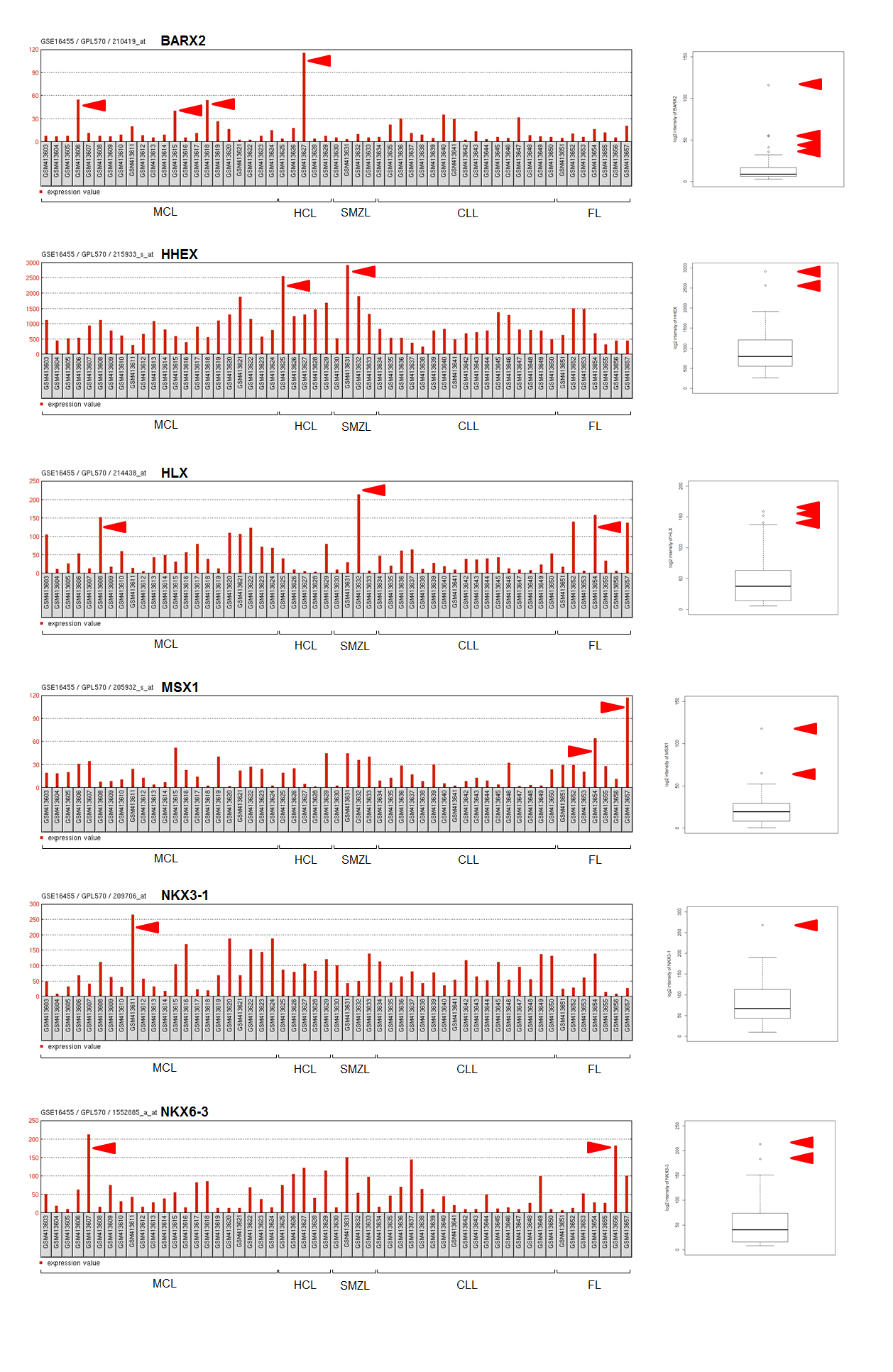

Supplement: S5 Fig — Expression profiling dataset GSE16455 contains patient samples for mantle cell lymphoma (MCL), hairy cell leukemia (HCL), splenic marginal zone lymphoma (SMZL), chronic lymphocytic leukemia (CLL), and follicular lymphoma (FL). We set the cutoff at 100 for positive gene activity. Boxplots were performed for expression levels and outliers were defined as aberrant overexpression, highlighted by red arrowheads. Accordingly, BARX2 and HHEX were each overexpressed in 1/5 (20%) of HCL patients. BARX2 was overexpressed in 3/22 (14%) of MCL patients. HHEX and HLX were each overexpressed in 1/4 (25%) of SMZL patients. HLX, NKX3-1 and NKX6-3 were each overexpressed in 1/22 (4%) of MCL patients. HLX and NKX6-3 were each overexpressed in 1/7 (14%) and MSX1 in 2/7 (28%) of FL patients. (TIF) [file pone.0205537.s005.tif]

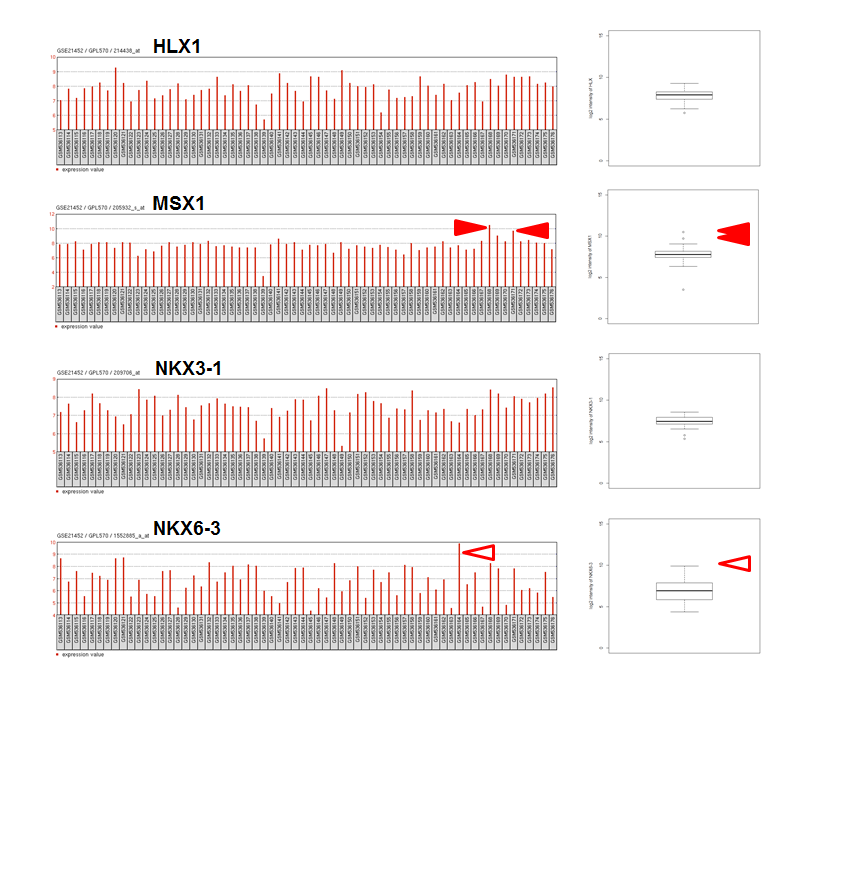

Supplement: S6 Fig — Expression profiling dataset GSE21452 contains 64 patient samples for mantle cell lymphoma. We defined a cutoff at 8 to reveal positive gene activity. Boxplots were performed for expression levels and outliers were defined as aberrant overexpression, highlighted by red arrowheads. Accordingly, MSX1 was overexpressed in 2/64 (3%) of MCL patients. Of note, in this dataset no overexpression of HLX and NKX3-1 was detectable–overexpression of NKX6-3 was not significant. (TIF) [file pone.0205537.s006.tif]

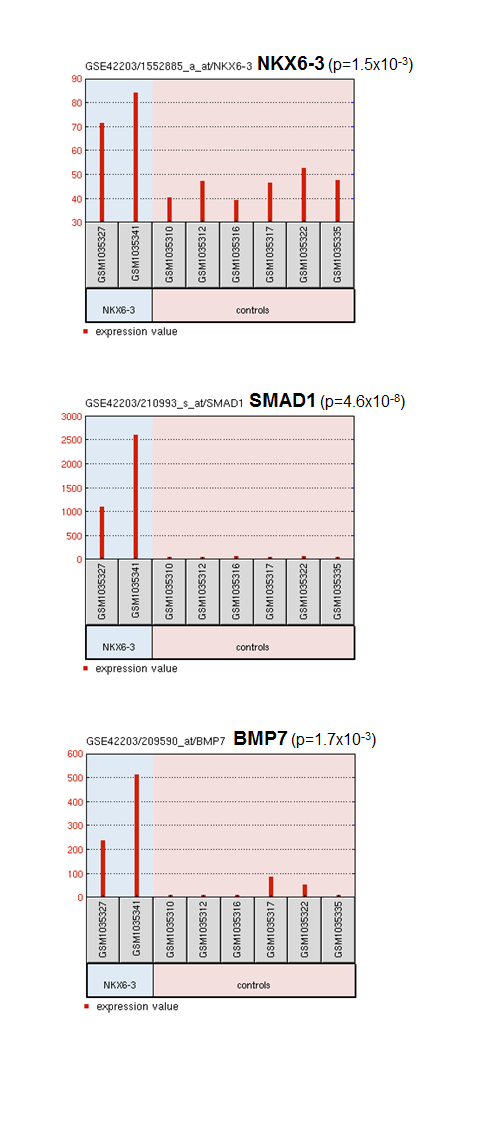

Supplement: S7 Fig — Expression profiling dataset GSE42203 contains two DLBCL cell line samples (DOHH-2 and OCI-LY1, blue background) showing overexpression of NKX6-3. Expression levels of NKX6-3, SMAD1 and BMP7 were shown in comparison to six control DLBCL cell lines (SU-DHL-16, SU-DHL-10, SU-DHL-8, SU-DHL-5, SU-DHL-4, SU-DHL-7, red background). The statistical significances are indicated as p-values. (TIF) [file pone.0205537.s007.tif]

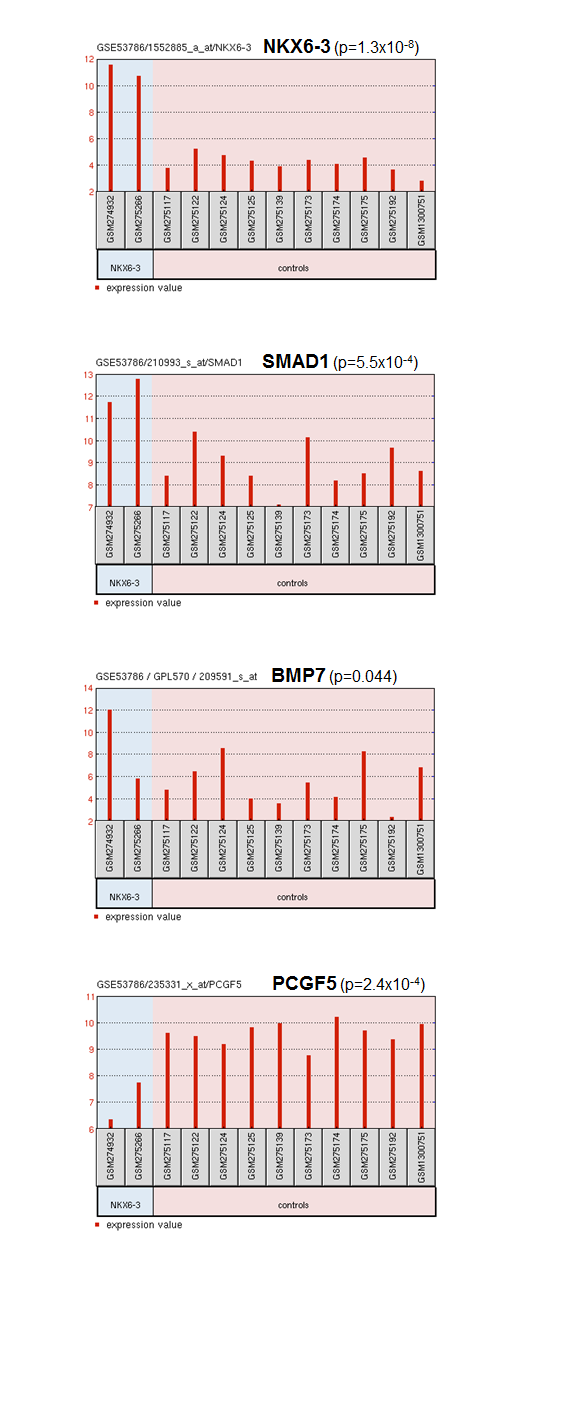

Supplement: S8 Fig — Expression profiling dataset GSE53786 contains two DLBCL patient samples showing overexpression of NKX6-3 (blue background). Expression levels of NKX6-3, SMAD1, BMP7 and PCGF5 are shown in comparison to 10 control DLBCL patients (red background). The statistical significances are indicated as p-values. (TIF) [file pone.0205537.s008.tif]

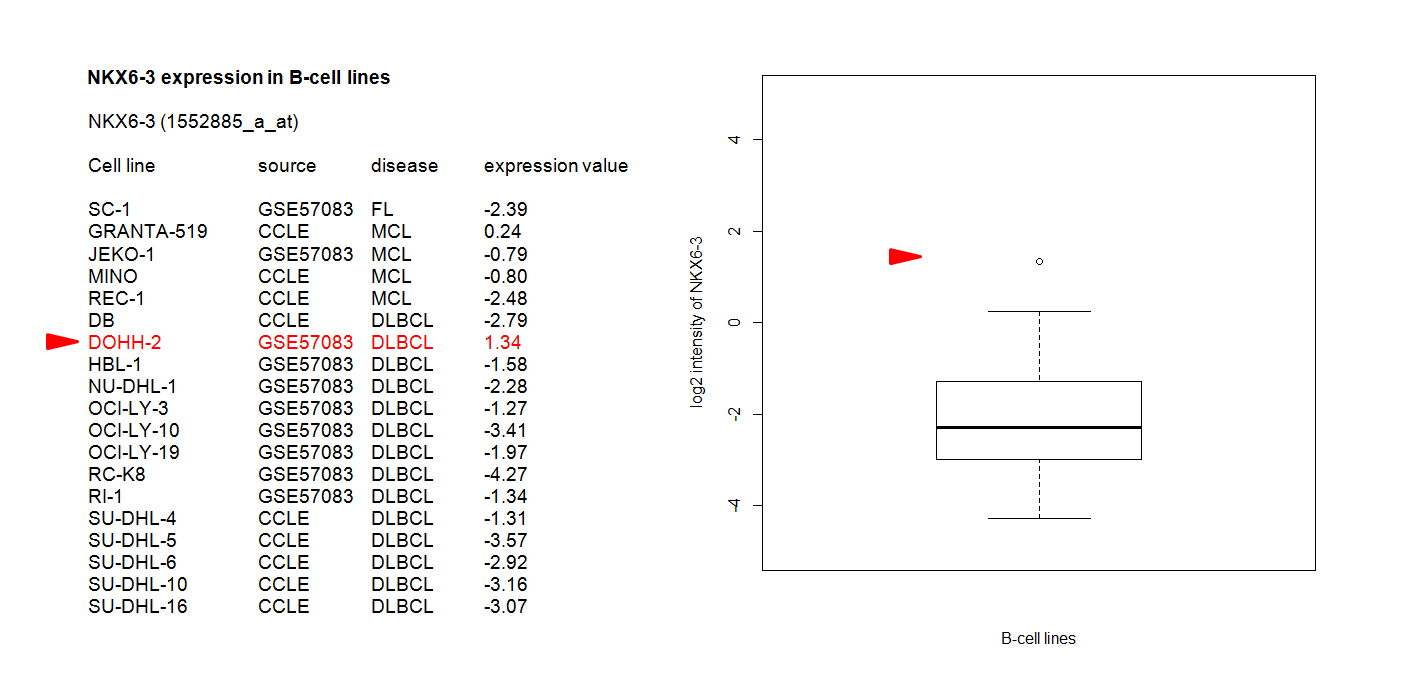

Supplement: S1 Table — Expression profiling data for NKX6-3 of 19 B-cell lines are indicated in addition to the source of data and the derived malignancy (left). Representation of the data in a boxplot demonstrates the value of DOHH-2 as outlier, demonstrating NKX6-3 overexpression in this cell line (right). (TIF) [file pone.0205537.s009.tif]
